# Nanomaterials targeting cancer stem cells to overcome drug resistance and tumor recurrence

**DOI:** 10.3389/fonc.2025.1499283

**Published:** 2025-06-06

**Authors:** Divya Vishambhar Kumbhakar, Lucky Thakkar, Chetana Akhand, Shehna Sharaf, Geeta K. Vemuganti

**Affiliations:** ^1^ Stem Cell and Cancer Biology Laboratory, School of Medical Science, University of Hyderabad, Telangana, India; ^2^ Thakkar Consultancy Services, Janjgir, Chhattisgarh, India; ^3^ Environmental Biotechnology and Genomics Division (SEP-EB), Council of Scientific and Industrial Research-National Environmental Engineering Research Institute (CSIR-NEERI), Nagpur, Maharashtra, India; ^4^ L V Prasad Eye Institute, Hyderabad, Telangana, India

**Keywords:** cancer stem cells, signaling pathways, biomarkers, nanocarriers, exosomes, nanoparticle-mediated ablation therapies, targeted therapy

## Abstract

A cancer stem cell (CSC) is an immortal cell that is capable of self-renewal, continuous proliferation, differentiation into various cancer cell lineages, metastatic dissemination, tumorigenesis, maintaining tumor heterogeneity, and resistance to conventional treatments. Targeted therapies have made huge advances in the past few years, but resistance is still a major roadblock to their success, in addition to their life-threatening side effects. Progressive treatments are now available, including immunotherapies, CRISPR-Cas 9, sonodynamic therapy, chemodynamic therapy, antibody–drug nanoconjugates, cell-based therapies, gene therapy, and ferroptosis-based therapy, which have replaced surgery, chemotherapy, and radiotherapy for cancer treatment. The challenge is to develop targeted treatment strategies that are effective in eradicating CSCs, as they are resistant to anticancer drugs, causing treatment failure, relapse, and recurrence of cancer. An overview of the fundamental characteristics of CSCs, drug resistance, tumor recurrence, and signaling pathways as well as biomarkers associated with their metastatic potential of CSC is elucidated in this review. The regulatory frameworks for manufacturing and conducting clinical trials on cancer therapy are explicated. Furthermore, we summarize a variety of promising nanocarriers (NCs) that have been used directly and/or synergistic therapies coupled with the therapeutic drug of choice for the detection, targeting, and imaging of CSCs to surmount therapeutic resistance and stemness-related signaling pathways and eradicate CSCs, hence alleviating the limitation of conventional therapies. Nanoparticle-mediated ablation therapies (NMATs) are also being argued as a method for burning or freezing cancer cells without undergoing open surgery. Additionally, we discuss the recent clinical trials testing exosomes, CRISPR/Cas9, and nanodrugs, which have already received approval for several new technologies, while others are still in the early stages of testing. The objective of this review is to elucidate the advantages of nanocarriers in conquering cancer drug resistance and to discuss the most recent developments in this field.

## 1 Introduction

The American Cancer Society estimates that cancer is the second leading cause of death in the United States, and the number of cancer cases is projected to increase to 2,041,910 in 2025, with cancer deaths expected to reach 618,120 (1). CSCs are vicious and can be a significant contributing factor to cancer treatment failure (2). Cancer stem cells (CSCs) are a small subset of cancer cells with regeneration capabilities and excessive tumorigenic potentials, involved crucially in tumor growth, progression, invasion, and metastasis (2, 3). Tumors are heterogeneous and contain both differentiated tumors and undifferentiated cancer stem cells (4). Numerous conventional modalities including surgery, chemotherapy, and radiotherapy are available for treating a wide range of malignancies (5, 6). Studies have revealed that CSCs have inherent drug resistance to conventional modalities as well as developmental plasticity (7, 8), allowing CSCs to differentiate into mature progeny (9). Moreover, differentiated cancer cells can undergo a stem cell-like transformation (10). Conventional treatments primarily target the tumor but often fail to eliminate drug-resistant CSCs due to the overexpression of anti-apoptotic proteins, ATP binding cassette (ABC) transporters, enhanced DNA damage response, elevated DNA repair, increased survival signaling, epithelial–mesenchymal transition (EMT) induction, epigenetic mechanism, hypoxia and low reactive oxygen species (ROS) level (11, 12), increased quiescence, increased autophagy, detoxifying enzymes (ALDH1), and signaling pathways (Wnt/β-catenin, Notch, hedgehog, Hippo, and PI3K/Akt, JAK/STAT), which led to drug resistance and tumor recurrence (12–15). The tumor tissue has an extracellular pH of 6.8 (acidic), favoring metalloproteinases, activating several signaling pathways, and serving as a blockade for many anticancer drugs that accentuate the malignancy and aggressiveness of cancer cells (16–18). Despite the availability of many treatment options, resistance to treatments still occurs, causing the cancer to recur, a phenomenon explained by CSC, imposing an innovative outlook for cancer treatment (11, 14).

The nanocarriers (NCs) used in cancer treatments are usually in the range of 20–200 nm, allowing them to circulate more quickly and absorb more readily into cells (19, 20). By virtue of their enhanced permeability and retention (EPR) effect, these NCs passively extravasate leaky tumor vessels and accumulate in tumors (21), allowing medications to be delivered to cancer cells and avoiding contact with healthy cells (21, 22). NCs are on the horizon as a novel breakthrough in targeted therapy. They provide altered therapeutic possibilities over conventional approaches and are impeccably able to modulate drug delivery and accumulate at target sites specifically to treat tumor-targeting CSCs (23–26). Biomedical researchers have increasingly embraced nanotechnology over the past decade and focused on the nanomaterial-loaded drug delivery (NDD) strategy targeting CSCs based on their markers (27), hence perceived by means of cell imaging, immunotherapy, multimodal synergistic therapies, siRNA delivery, and targeted cancer therapy (28–30). NDD via endocytosis bypasses the efflux pump, resulting in intracellular accumulation in CSCs (31, 32). The co-delivery of anticancer drugs, multiple drug resistance modulators, and CSC-targeting ligands using NDD could boost the specificity of CSC to surmount drug resistance (33–35). In addition to providing a comprehensive understanding of CSCs, our goal is to present a summary of recent cancer nanotherapies, both basic and applied, as well as new treatments that are currently being researched and hoped to overcome conventional treatment limitations. As innovative anticancer strategies, different approaches to diagnosis and therapy will be discussed, highlighting their current status in the clinical context. In this review, we introduce the treatment modalities, involving drug-loaded inorganic NCs, antibody–drug conjugates polymer-based NCs, self-assembling protein NCs, exosomes, and MXene, which have been reported to interact with tumor-associated stem cells, as well as with CSC-related signaling pathways, and are being used as diagnostic and therapeutic agents. The review describes the advances in technologies to reduce CSCs, including photothermal therapy (PTT) and nanoparticle-mediated ablation therapies (NMATs), and bioengineered exosomes’ role in antitumor therapies in order to encounter the prevailing complications of therapy resistance. Understanding the merits and limitations of these treatments offers new perspectives for clinical practice and groundbreaking research.

## 2 Cancer stem cells

### 2.1 Characteristics

A number of studies suggest that CSCs not only are responsible for tumor growth, maintenance, and resistance to chemotherapy and radiotherapy but also contribute to cancer recurrence after treatment since they can regenerate the tumor (10, 14, 36). However, non-CSCs are more differentiated and less likely to cause tumor growth or recurrence (37). CSCs may be resistant to therapy by activating survival pathways, remaining in quiescent states, increasing drug efflux, impairing apoptosis, and repairing DNA damage more efficiently (11, 12). A significant feature of CSCs is their capability to modify the surrounding stroma by secreting proteins and molecular components such as extracellular matrix (ECM) proteins, which helps in maintaining the CSCs in a dormant state to regulate their fate, plasticity, and resistance against conventional therapies (37–39). Their self-renewal capacity can lead to uncontrolled differentiation with transformed cellular and molecular phenotypes, resulting in the formation of heterogeneous primary and metastatic tumor cells that are resistant to treatment and contribute to tumor recurrence (38, 40). The major characteristics of CSCs include (2, 41–45) the following:

- self-renewal and differentiation properties,
- presence of specific surface markers for identification,
- ability to generate after transplantation,
- resistance to chemotherapy and radiotherapy,
- initiation of a new tumor through pre-existing CSCs,
- altered expression of transcription factors, receptors, and signaling pathways,
- ability to divide symmetrically into two CSCs or one CSC and one daughter cell,
- ability to thrive in hypoxic microenvironments,
- plasticity, which is the ability to adapt to new environments following phenotypic transition, and
- increased mobility, migratory, and invasive properties.

### 2.2 Chemoresistance of drug and self-renewal ability

The Darwinian notion of survival of the fittest applies to cancer cells attaining drug-resistant traits at molecular levels for survival (12, 39, 46, 47). Numerous *in vitro* and *in vivo* examinations have shown that conventional therapies induce CSCs, which later contribute to tumor relapse and therapy resistance (48). CSCs depend on multiple pathways for chemoresistance and self-renewal (10, 49). Thus, targeting these pathways can guide us to a strategic mechanism to overcome resistance. Notch1 signaling plays a key role in enhancing trastuzumab resistance in breast cancer cell lines BT474, SK-BR3, and MCF-7 cells; its inhibition, either genetic or pharmacological, enhances the sensitivity of these cells to the drug, i.e., making them more responsive to the drug’s effect (12). The Notch activity was boosted in both bulk and breast cancer stem-like cells in ER+ and HER2+ breast cancer cell lines upon treatment with tamoxifen or trastuzumab drugs, respectively (50). Knocking down Notch triggers significant growth arrest in these cells, leading to loss of stem-like characteristics such as self-renewal, tumor recurrence, resistance to drugs, and EMT (50). Significant molecular alteration was observed in breast cancer upon treatment of γ-secretase inhibitors, i.e., tamoxifen or letrozole, i.e., an aromatase inhibitor (reversible non-steroidal imidazole-based inhibitor) (12). As a first-line treatment for glioblastoma multiforme (GBM), humanized monoclonal anti-VEGF antibodies (bevacizumab) were effective in reducing tumor formation (12). The clinical benefit, however, lasted for a short time due to the development of resistant lineages and the dominance of VEGF-VEGFR2-Neuropilin-1 autocrine signaling over time, resulting in tumor relapses (51, 52). Glioblastoma CSCs (CD133^+^/Prominin-1) induced by radiotherapy can increase resistance by activating DNA checkpoints and repair pathways. Therefore, co-treatment with checkpoint inhibitors (Chk1 and Chk2) and radiotherapy increased the radiosensitization of glioblastoma CSCs (53). CSCs often confiscate pluripotent or oncofetal drivers, as they share critical features of embryonic stem cells for the expression of transcriptional factors such as SALL4, NANOG, KLF4, MYC, OCT4, and FOXM1 and signaling pathways such as Hedgehog, Notch, Hippo, Wnt/β-catenin, and TGF-β (54, 55). Lin28B (RNA-binding proteins that affect stem cell maintenance, metabolism, and oncogenesis) has been identified as an oncofetal circulator CSC marker and a crucial therapeutic target for hepatocellular carcinoma recurrence (56, 57). These oncofetal stem cell markers are not expressed by normal stem cells, so they serve as prime targets for therapy (12). Multiple cellular processes such as increased DNA damage and repair, entering into a dormant state, quick drug efflux, and anti-apoptotic protein overexpression, are mechanisms that lead to drug resistance (58, 59). Therefore, the removal of CSCs has become a prime target among the scientific fraternity. Nanocarriers have been proven a promising tool to deliver chemotherapeutic drugs at high dosages and release them to their target to control the CSCs, leading to overcoming the resistance and recurrence of CSCs (58).

### 2.3 CSC markers and challenges encountered in biomarker identification

Different cancers have distinct molecular and genetic profiles, which influence the markers expressed by CSCs (60). CSC markers mimic those of normal stem cells, resulting in difficulties in differentiated and targeted CSCs. CSC markers express differently in diverse microenvironments including inflammation, hypoxia, and cell–cell interaction, prompting the CSC features and marker expression (61). CSCs are also capable of sustaining genetic and epigenetic changes, which can alter their marker properties, depending on the mutational tumor sites and their evolutionary pathway (62, 63). In order to ensure a successful therapy, somatic stem cells (SSCs) should not experience any side effects; if we understand how CSCs and SSCs differ in their origin, self-renewal mechanism, and signaling pathways, we will be able to target CSC populations more effectively, protecting healthy cells and minimizing side effects.

**Figure 1:**
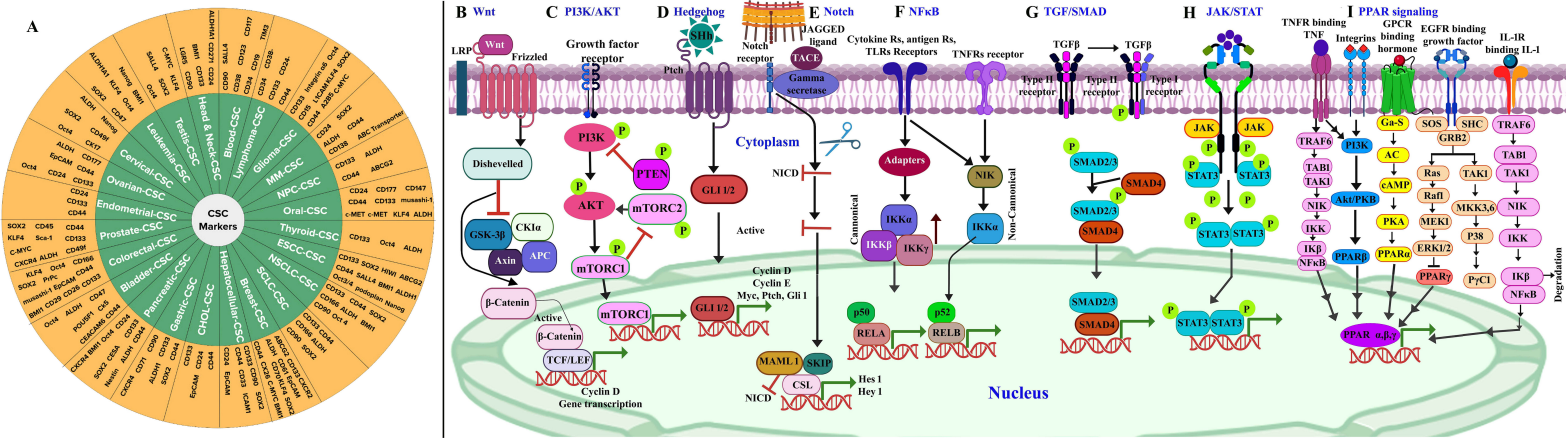
**(A)** Cancer stem cell markers in different types of cancer (13, 104–110). A schematic illustration of signaling pathways involved in cancer stem cells. **(B)** Wnt/β-catenin: targets include Wnt/Frizzled complexes, β-catenin/TCF, and CK1α. **(C)** PI3K/AKT: targets include PI3K complex, AKT1/2/3, and mTORC1/2. **(D)** Hedgehog: targets include SHh-Ptch interaction, SMO, and GLI. **(E)** Notch: targets include Notch and γ-secretase. **(F)** NF-κB: targets include IKKα/β/γ and NF-κB-inducing kinase (NIK). **(G)** TGF/SMAD: targets include TGF-β1/β2/β3, TβRI/II, and Smad3/4/5. **(H)** JAK/STAT: targets include JAK1/2/3 and STAT1/2/3/4/5. **(I)** PPAR: targets include PPARα/γ/δ signaling pathways. Adapted and modified using BioRender for illustrative purposes with permission from Chu et al. (2) (Copyright 2024).

CSC targeting strategies have proved to be difficult due to phenotypic plasticity in tumors, which allows non-CSCs to acquire CSC traits, complicating CSC targeting strategies (39, 64). This necessitates the use of specific cell surface markers detected on CSCs for better results (38). Different markers can be expressed by CSCs depending on the tissue from which they originate (4, 11). Some of the markers associated with CSCs are cell surface markers, signaling pathways, transcription factors, and drug transporters, as well as genes, proteins, enzymes, and miRNA, which are responsible for self-renewal, immune evasion, metastasis, and treatment resistance (65) (**Table 1**, **Figure 1A**). The CSC-specific surface markers include CD24, CD26, CD44, CD133, CD166, aldehyde dehydrogenase (ALDH), and Ep‐CAM (also called CD326 or epithelial‐specific antigen/ESA) (61, 111, 112). The markers like CD24, CD34, CD44, CD133, CD166, and ALDH1 were used for the identification of CSCs in solid bulk tumors (61, 112). Common stem cell markers include CSC-specific markers such as CD34, CD44, CD123, CD133, c-kit, ABCG2, and ALDH, which have been reported in a wide range of malignancies (113, 114). CSCs are also intrinsically regulated by stemness-related transcription factors, such as OCT-4, SOX2, KLF4, c-MYC, STAT3, and NANOG, as well as epigenetics and epi-transcriptomics, which are important for stemness maintenance and plasticity (112). **Figure 1A** presents markers specific to cancer stem cells in different types of cancer (13, 104–110), and these markers are primarily useful for targeting CSCs for therapeutic purposes.

**Table 1:** CSC biomarkers and their diverse role in various cancer types.

Biomarkers	Type of cancer	Role of CSCs	References
CD44^+^	Ovarian cancers, breast cancer, colon cancer, gastric cancer, prostate cancer, lung cancer	Cell survival, cellular motility, cell–cell interactions and signaling, cell proliferation, EMT regulation, cytoskeletal changes, stemness, tumor metastasis and progression	(34) (66) (67) (68) (69) (70)
CD133^+^	Kidney cancer, brain cancer, liver cancer, pancreatic cancer, colon cancer, gastric cancer, lung cancer, breast cancer, cervical cancer, prostate cancer	Tumorigenesis, metastasis, tumor recurrence, therapeutic resistance, mainstream lung cancer marker	(67) (68) (70) (71) (72) (73) (74)
CD90^+^	Gastric cancer, liver cancer, esophageal squamous carcinoma, lung cancer, skin cancer, brain cancer, pancreatic cancer	Cell proliferation, metastasis, angiogenesis, prognostic marker, cell–cell and cell–matrix interactions	(68) (75) (76)
CD166^+^	Gastric cancer, ovarian cancer, prostate cancer, breast cancer, head and neck cancer, liver cancer, lung cancer, melanoma, colorectal cancer, esophageal cancer, bladder cancer	Metastasis, apoptosis evasion, cancer initiation, invasiveness, melanoma cell clustering, activation of tumorigenic signaling pathway, cell adhesion, tumor progression, hematopoiesis	(68) (77) (78) (79) (80) (81)
CD24^+^	Liver cancer, ovarian cancer, prostate cancer, breast cancer, esophageal squamous cell carcinoma, lung cancer, pancreatic cancer	Cancer progression, tumorigenesis, tumor evasion, cell proliferation and invasion, prognostic and diagnostic marker, tumor resistance and tumor recurrence, metastasis, immune evasion	(75) (82) (83) (84)
NANOG	Ovarian cancers, liver cancer, breast cancer, lung cancer, colorectal cancer, leukemia, prostate cancer, brain cancer, gastric cancer, head and neck cancer, pancreatic cancer, cervical cancer	Metastasis, chemoresistance, stemness, invasiveness, self-renewal, tumorigenesis, prognostic marker, initiation and sustainability of tumor, drug resistance, pluripotency	(34) (85) (86) (87) (88)
OCT4	Ovarian cancers, gastric cancer, glioma, acute myeloid leukemia, bladder cancer, prostate cancer, rectal cancer, melanoma, liver cancer, esophageal squamous cell carcinoma	Tumorigenesis, tumor progression, stemness, pluripotency, cancer stem cell maintenance, chemoresistance, drug resistance, stem cell differentiation and self-renewal, angiogenesis	(34) (68) (89) (90) (91)
SOX2	Ovarian cancers, gastric cancer, head and neck cancer, lung cancer, breast cancer, medulloblastoma, skin cell carcinoma, bladder cancer, pancreatic cancer, cervical cancer, colorectal cancer	Prognostic marker, stemness, self-renewal, cell proliferation, drug resistance, tumor initiation, progression and aggressiveness, metastasis, EMT enhancement, therapeutic target, prospective biomarker, tumorigenicity, chemoresistance	(34) (68) (86) (92) (93) (94)
EpCAM	Liver cancer, colorectal cancer, breast cancer, ovarian cancer, pancreatic cancer, gallbladder cancer, thyroid cancer, endometrial cancer, lung cancer	CSC self-renewal and differentiation, tumor progression and survival, chemotherapeutic resistance, therapeutic strategy, molecular biomarker, cell adhesion and migration, cell–cell interactions, prognostic marker, angiogenesis, tumorigenicity, cancer initiation	(75) (95) (96) (97) (98)
ALDH1A1	Lung cancer, ovarian cancer, breast cancer, liver cancer, esophageal cancer, gastric cancer, cervical cancer, stomach cancer, pancreatic cancer, thyroid cancer, prostate cancer,	Tumor initiation, progression, invasion, and migration, cancer cell proliferation, adhesion, extravasation, micrometastasis, self-renewal and differentiation, drug and chemoresistance	(70) (77) (99) (100) (101)
LGR5	Gastric cancer, colon cancer, colorectal cancer, glioblastoma, breast cancer, ovarian cancer, adenocarcinoma, thyroid cancer	CSC identification, cancer initiation, recurrence and therapeutic resistance, tumor initiation, progression, metastasis, CSC proliferation and self-renewal, EMT, CSC biomarker	(68) (86) (102) (103)

CSC, cancer stem cell; EMT, epithelial–mesenchymal transition.

### 2.4 CSC signaling pathways and FDA-approved drugs as inhibitors

**Figure 2:**
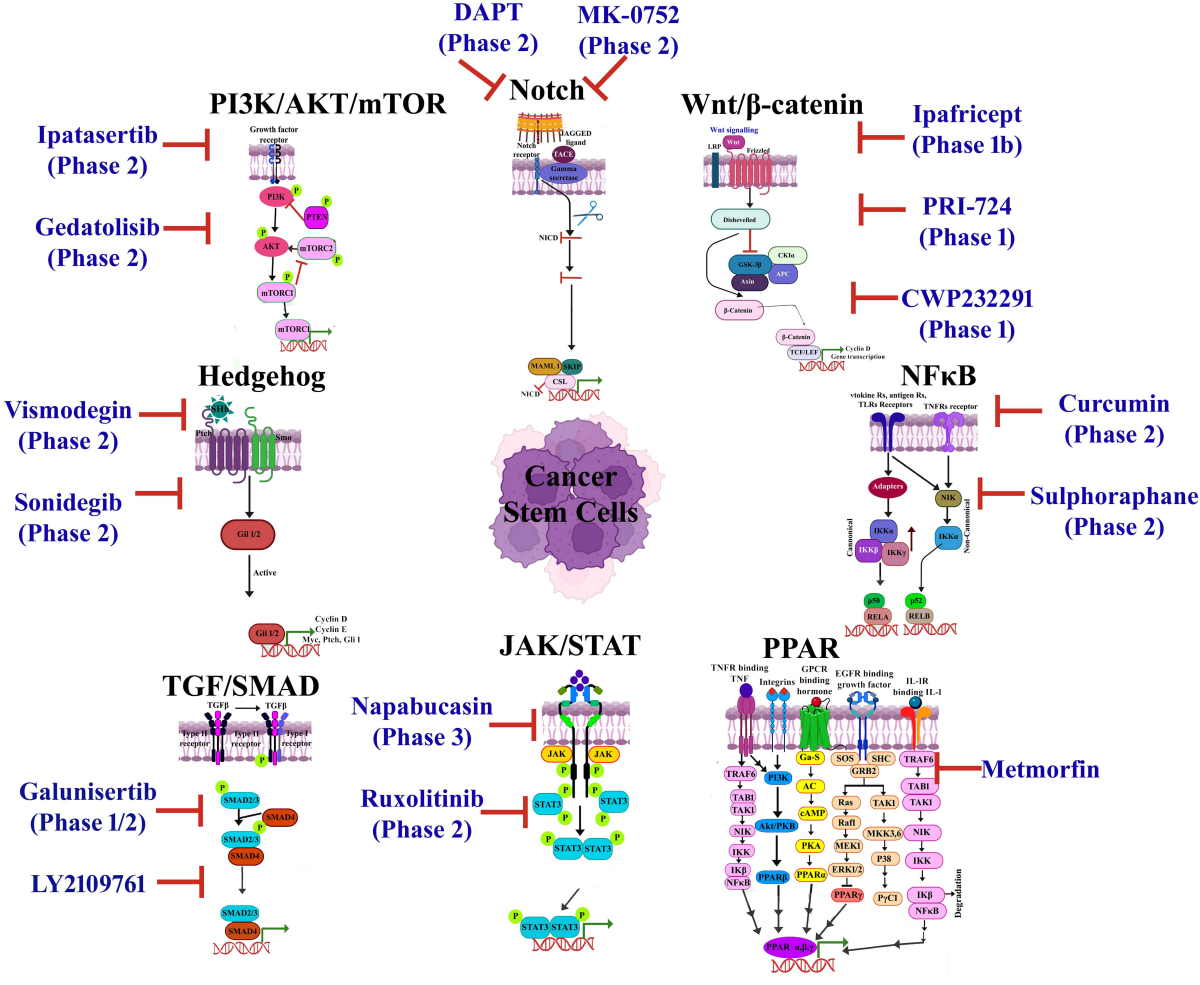
An overview of a few cancer stem cell (CSC)-targeted therapeutic agents that have been approved or are undergoing clinical trials. Adapted and modified with permission from PS Kharkar (Copyright 2020, American Chemical Society) (45, 119–123).

The recurrence of CSCs is due to their resistance to existing conventional therapies, along with their high potential for metastasis and invasiveness (115). CSC signaling pathways are aberrantly activated in cancer, which govern self-renewal, cell proliferation, invasion, metastasis, and angiogenesis (44). The CSC transformation from a normal cell is due to accretions of genetic alterations, tumor suppressor genes, epigenetic modification [including (epi)methylation, demethylation, mutations, and rearrangements in the stem/progenitor pool (niche) and differentiated cells], and tumor microenvironment stimulation through extracellular signals (61, 112) A new challenge in cancer treatment is selecting the signaling networks that facilitate self-renewal, proliferation, and differentiation in CSCs that regulate tumorigenesis process. Most common CSCs associated with oncogenic cascades comprise Wnt/β-catenin (**Figure 1B**), phosphoinositide 3 kinase (PI3K)/AKT/mTOR (**Figure 1C**), hedgehog (**Figure 1D**), Notch (**Figure 1E**), NF-κB (**Figure 1F**), TGF-β/SMAD (**Figure 1G**), JAK/STAT (**Figure 1H**), and peroxisome proliferator-activated receptors (PPARs) (**Figure 1I**) (61, 116, 117). The effectiveness of small molecule inhibitors in cancer treatment is still challenged by minimal and short response values/duration, systemic toxicity, CSC biomarkers, and drug resistance (118). Currently, the Food and Drug Administration (FDA) has approved approximately 88 small molecule inhibitors for the treatment of cancer after clinical trials (118). The inhibitors that underwent clinical trials and target major pathways are implicated in the CSC pathway (**Figure 2**).

#### 2.4.1 Wnt/β-catenin

The Wnt/β-catenin signaling cascade plays a key role in CSC biology, leading to self-renewal, uncontrolled cell proliferation, and differentiation (124). The dysregulation in Wnt/β-catenin signaling has been documented in a wide range of malignant cancers such as leukemia, colon, epidermal, breast, and cutaneous carcinoma (125, 126). Numerous methodologies have been upgraded for targeting Wnt/β-catenin cascade involving small molecules (ICG-001, PRI-724, E7386), which inhibit the interaction between TCF/LEF1 and β-catenin, thereby interrupting self-renewal property of CSC (124). Recent investigations used monoclonal antibodies (mAbs) against Wnt ligands and their subsequent receptors as a target for CSC-based therapy (127). The Wnt/B catenin signaling pathway targets a wide range of small molecules, including Wnt974, Wnt-C59, ONC201, Niclosamide, XAV939, Chelerythrine, FH535, IWR-1, IC-2, JIB-04, DTX and SFN, PP, OXT-328, AD, and Ts (**Figure 2**) (45, 128–135). Specifically, these compounds inhibit CSC progression/population, suppress self-renewal ability, attenuate CSC-mediated chemoresistance, and deregulate CSC markers and genes, resulting in drug resistance and compounding its sensitivity (45, 136–138).

#### 2.4.2 Hh signaling

Hh signaling contributes significantly to various stages of cell development; mutation at any stage of the sonic hedgehog (Shh) pathway can lead to the advancement of numerous cancers such as melanoma, rhabdomyosarcoma, medulloblastoma, and basal cell carcinoma, as well as breast, pancreas, lung, liver, and prostate cancers (58, 139). Aberrant Hh signaling promotes CSC self-renewal and resistance to treatment and its hyperactivation (mutations/deregulation), which leads to tumorigenesis (41). Studies have shown that inhibiting the aberrantly active Hh pathway in non-small-cell lung cancer (NSCLC) using a Hh antagonist led to a significant reduction in cell viability and malignancy (140). CSC progression can be inhibited by small molecules including glasdegib, sonidegib, vismodegib, ciclesonide, cyclopamine, and GANT61 by suppressing Hh signaling (45, 64, 141–145) (**Figure 2**). These small molecules inhibit CSC marker expression, self-renewal and mammosphere formation, and CSC proliferation and survival (45, 64, 141–145).

#### 2.4.3 Notch signaling

Notch signaling regulates cell-to-cell communication right from embryogenesis, cellular proliferation, differentiation, and even in apoptosis (146), also crucial for neural stem cell survival, immune regulation, colorectal epithelial maturation, breast development, and normal hematopoiesis (41). The Delta-like ligand 4 (Dll4) is one of the Notch signaling ligands that contribute to malignancy progression (147). There have been numerous reports of mutations of the Notch gene, including those of Dll4, which have been implicated in the growth of different types of gynecological tumors (148). Inhibitors of Notch act on N1ICD, γ-secretase, Hes-1, Hey-1, and Notch ligands to treat cancer and prevent recurrence (45, 149). Several pharmaceutical drugs, including MK-0752, PF-03084014, RO4929097, DAPT, and Quinomycin A (**Figure 2**) reduce the mammosphere formation, strike tumor regeneration, impede CSC growth, induce CSC differentiation, and decrease drug resistance in reverse, increasing drug sensitivity (45, 149–152).

#### 2.4.4 TGF-β/SMAD

The tumor cells secrete interleukin-33 cytokine, which causes myeloid cell differentiation into macrophage and consequently stimulates TGF-β signals to reach cancer stem cells, resulting in the progression of malignant tumors and drug resistance (153). TGF-β serves as a significant target commonly for multiple malignant tumors (breast, lung, liver, colon, among others) and found to be involved in the initial developmental stage and maintenance of CSCs (154, 155). Few inhibitors that address the unmet clinical necessities in cancer immunotherapies targeting TGF-β/SMAD signaling pathway are galunisertib (LY2157299) (156), vactosertib (TEW-7197) (157), LY2109761 (158), LY3200882 (159), MDV6058 (PF-0695229), GFH018 (160), YL-13027 (157), AGMB-129 (ORG-129) (161), SH3051, Trabedersen (AP 12009) (157), fresolimumab (GC1008) (162), AVID200 (163), ABBV-151 (164), SRK-181 (165), and bintrafusp alfa (M7824) (166). The inhibitor shown in **Figure 2** blocks the TGF-β/SMAD pathway (164, 167–172).

#### 2.4.5 PPAR

The PPAR pathway activation (comprising PPARα, PPARδ, and PPARγ subtypes) involves the binding of G-protein-coupled receptors to its respective ligand, which leads to the induction of translocation of nuclear receptor protein PPAR responsible for gene expression (173–176). The PPARs are involved in cell proliferation modulation, apoptosis, cell survival (stimulatory or inhibitory effects on cancer progression), EMT process regulation, and stem cell-like properties of CSCs (116). PPARβ/δ also regulates tumor angiogenesis *in vivo* and *in vitro* in CSCs by the promotion of proangiogenic factors such as VEGF and interleukin-8 (IL-8) (177). PPARα and PPARβ/δ regulated CSCs for metabolic reprogramming in GBM, lung cancer, and mouse mammary gland carcinoma, suggesting its association with CSC metabolism (178). A clinical trial is being conducted with efatutazone and metformin, which target the PPAR signaling pathway (**Figure 2**) and have antiproliferative, anticancer stem cell activity, maintenance of chemosensitivity, apoptosis, and reverse chemotherapy resistance and reduce migration and metastasis of cancer cells (179, 180).

#### 2.4.6 JAK/STAT

JAK/STAT signaling plays a crucial role in the development of multiple cancers and is directly associated with growth, metastasis, and progression whereas indirectly linked to the immune surveillance modulation and is activated during the recruitment and activation of JAK by the cytokine receptors (181, 182). The receptor tyrosine is then phosphorylated by JAK followed by the recruitment of STAT proteins (182, 183). The phosphorylation of STAT results in the translocation of its dimers to the cell nucleus for DNA binding to initiate the transcription of target genes (184). The JAK protein consists of JAK1–3 and Tyk2, whereas the STAT family comprises STAT5a, STAT5b, STAT1-4, and STAT6. In the case of high-grade gliomas, JAK1/2-STAT3 along with a hypoxia-induced pathway utilizing hypoxia-inducible factor 1α (HIF-1α) TF has been reported for enhancing the self-renewal capability of glioma stem-like cells (178, 181, 185). The antitumor molecules Pacritinib, fedratinib, tofacitinib, baricitinib, abrocitinib, filgotinib, oclacitinib, peficitinib, upadacitinib, deucravacitinib, and delgocitinib (186–193) have garnered interest as potential candidates for modulating the JAK/STAT pathway (**Figure 2**) and were found to be effective in reducing cell proliferation and viability, promoting apoptosis and obstructing invasion (189, 194).

#### 2.4.7 PI3K/Akt

PI3K/Akt is an intracellular phosphatidylinositol kinase, while the mTOR pathway comprises a regulatory subunit p85 along with a catalytic subunit p110 having serine/threonine (Ser/Thr) kinase and phosphatidylinositol kinase (195, 196). The three isoforms of Akt (Akt1-3) are directly activated by PI3K (197). The mTOR complex is a downstream target gene with two multiprotein complexes (mTORC1 and mTORC2) (198, 199). mTORC2 phosphorylates the Ser473 residue of Akt (200), resulting in Akt activation (178). This pathway can be activated by various mechanisms including insulin-like growth factor (IGF)/IGFR, ErbB, and fibroblast growth factor (FGF)/FGFR signaling (116, 201). The PI3K/AKT/mTOR pathway is crucial for the growth of cancer cells, involved in the cell cycle, proliferation, quiescence, migration and invasion of CSCs, and therapeutic resistance (178, 202, 203). Clinical analysis of multiple small molecules dysregulating PI3K/AKT/mTOR pathway, including buparlisib (BKM120), pictilisib (GDC-0941), idelalisib, alpelisib (BYL719), serabelisib, taselisib (GDC-0032), gedatolisib (PF05212384), voxtalisib (SAR245409/XL765), MK2206, capivasertib (AZD5363), perifosine, uprosertib (GSK-2141795), aspirin, rapamycin, everolimus, temsirolimus, metformin, onatasertib (CC223), sapanisertib, and vistusertib (AZD2014) (204–207) (**Figure 2**) are found to reduce tumor progression and improve chemotherapy treatment efficacy (46, 47, 208–219).

#### 2.4.8 NF-κβ

NF-κβ is a rapid inducible TF along with five different proteins, namely, RelB, NF-κβ1, NF-κβ2, p65, and c-Rel (220). The major physiological function of NF-κβ is p50-p65 dimer (221). The activity of NF-κβ complex is regulated by canonical and non-canonical signaling pathways (220, 222). Cytokines involved in tumor-promoting inflammation including TNF-α, IL-1, IL-6, COX2, iNOS, and MCP1, and factors like Cyclin E, Cyclin D, and proto-oncogen c-Myc are accountable for the activation of the NF-κβ pathway resulting in the cancer cell proliferation (160, 178, 223, 224). The NF-κβ pathway is involved in the stimulation, EMT, invasiveness, angiogenesis, apoptosis prevention, and metastasis of CSCs (160, 184). The NF-κB signaling cascade is reported to be targeted using ferulic acid, vanillic acid, curcumin, resveratrol, nobiletin, trilobatin, apigenin, cirsiliol, scutellarein, acacetin, chalcone 2, luteolin, anthocyanidin, ginsenoside Rg-3, chlorogenic acid, quercetin, dehydroxymethylepoxyquinomicin (DHMEQ), nepalolide A, and parthenolide (**Figure 2**), ensuring interruption in tumor growth and proliferation, considering low toxicity to healthy cells (224–239).

## 3 CRISPR/Cas9 technology for cancer therapy

CRISPR/Cas9 (Clustered regularly interspaced short palindromic repeat)/CRISPR-associated protein 9) is a revolutionary genome-editing tool that can be used to regulate endogenous gene expression by both gene insertion and knockout relying on the Cas9 protein and the guide RNA (gRNA), making it a very powerful and versatile tool (240, 241). The suppression of oncogenes or upregulation of tumor suppressor genes can improve targeted therapy by confronting drug resistance and improving immunotherapy with CRISPR/dCas9 (242). This tool has been used to treat a wide variety of cancers and has demonstrated prominent outcomes (243–246). Researchers using CRISPR/Cas9 technology have recognized novel genes for cancer treatment such as suppression of NAD kinase (NADK activates pentose phosphate pathway involved in cancer survival) or ketohexokinase (KHK suppression leads to elevated fructose metabolites intricate in liver cancer progression) and inhibit tumor growth (245). Chen and co-authors discovered that alisertib is more effective when HASPIN (histone H3-associated protein kinase) is inhibited through CRISPR/Cas9 in breast cancer (247). Protein-l-isoaspartate (d-aspartate) *O*-methyltransferase (PCMT1) promotes ovarian carcinogenesis through FAK-Src activation (248). Accordingly, many tumor-associated genes (oncogenes, drug-resistant genes, tumor suppressor genes, immune evasion genes, and metabolic reprogramming genes) are targeted through CRISPR-Cas9, for instance, KRAS, p53, EGFR, PTEN, Nestin, BRAF, HASPIN FGFR, FAK, BRCA gene, PIK3CA, VEGFR, HER2, LDHA, NADK, ALK, NOTCH1, PD-L1, ABCB1, TERT, and LGALS2 (190, 242, 245, 247–254). In 2016, Sichuan University’s West Society China Hospital recruited its first patient to test the effectiveness of CRISPR/Cas9 in cancer therapeutics (255). The use of CRISPR-edited T cells in a phase I clinical trial in patients with non-small-cell lung cancer has been demonstrated to be safe in human subjects with advanced non-small-cell lung cancer (256). The most significant barrier to clinical CRISPR/Cas9 applications is the lack of efficient and safe delivery systems (242). The delivery system must overcome many physical barriers, in addition to high encapsulation and biocompatibility, to deliver CRISPR/Cas9 components to the target, thereby attaining precise and effective treatment (242). There has been increasing attention to the application of non-viral vectors that rely on nanotechnology for anticancer cargo delivery (257). In addition to polymers, lipids, porous silicon, and mesoporous silica, have been used to treat different cancers because of their low immunogenicity, high biocompatibility, and ideal cargo delivery capabilities (242). Zhen et al. (258) injected nude mice with long-circulating pH-sensitive cationic liposomes targeted to splicing HPV16 E6/E7 cervical cancer cells, causing them to undergo apoptosis by inactivating them, thus inhibiting tumor growth without causing significant toxicity (258). In another study, multistage delivery nanoparticle (MDNP)/dCas9-miR-524 was administered to mice bearing MDA-MB-231 and LN-229 tumors, resulting in the significant upregulation of miR-524 expression (259). This upregulated expression then interferes with multiple signaling pathways associated with tumor proliferation, causing significant tumor growth retardation. MDNP was used to deliver CRISPR/Cas9, providing optimal efficiency in communicating with tumor tissues even in the face of multiple physiological barriers (259). Liu and colleagues discovered a nanoCRISPR system based on semiconductor polymers (SPs) that enables near-infrared (NIR) photoactivatable gene editing to advance the delivery proficiency of CRISPR and to improve cancer treatment effectiveness (259). This nanoCRISPR system can deliver sgRNA and generate heat using the photothermal effect when the NIR laser is irradiated (259). A localized heat event causes the dissociation of single-stranded DNA from single-stranded RNA to trigger sgRNA release, allowing precision cancer therapy using CRISPR (260).

### 3.1 Clinical trials of the CRISPR/Cas9 system for cancer therapy

We focused on published and ongoing clinical trials involving the CRISPR/Cas9 system’s capability of treating cancer. Phase I trials involving TALEN and CRISPR/Cas9 targeting HPV16 and HPV18 E6/E7 identifiers are underway to evaluate the safety and efficacy of the treatment for patients with HPV (+) CIN (261). In parallel, NCT04976218 specifies a phase I trial to evaluate CAR-EGFR-TGFR-KO T cells engineered through CRISPR/Cas9 to target TGF-β receptor II in previously treated EGFR-positive tumor cells (262). The CRISPR/Cas9 technology was used to knock out CD5 in CT125A cells (NCT04767308), a novel CAR T-cell therapy currently being tested in patients with relapsed/refractory CD5^+^ hematopoietic malignancies (263). The knockout of the PD-1 and TCCR genes using CRISPR/Cas9 was evaluated for safety, feasibility, *in vivo* persistence, and antitumor response in multiple solid tumor patients with mesothelin-positive cells (264). NCT03747965 is also associated with the CRISPR-engineered PD-1 gene knockout mesothelin-targeting CAR T-cell therapy for the treatment of neoplastic mesothelin-positive tumors in colorectal cancer (265). A trial evaluating the safety of PD-1 knockout T cells in patients with advanced esophageal cancer was completed and registered with identifier NCT03081715 (266). An alternative study identified as NCT05066165 aims to evaluate the activity and safety of NTLA-5001 in patients with acute myeloid leukemia following first-line or later treatment (267). NCT04035434 aims to investigate the safety and effectiveness of allogenic CRISPR/Cas9-engineered CTX110 T cells in patients with relapsed or refractory B-cell malignancies (268). C70-directed allogeneic CRISPR/Cas9-engineered CAR T-cell (CTX130) therapy in relapsed or refractory T-cell malignancies is being evaluated in another phase I study (NCT04502446) (269). Using premade allogeneic T cells from healthy donors (NCT05037669), a phase I study aims to evaluate the feasibility and safety of administering premanufactured allogeneic T cells that express CD19-targeting CAR knockouts targeting HLA class I, HLA class II molecules, and endogenous TCRs via CRISPR gene editing of beta-2 microglobulin, CIITA, and the T-cell receptor alpha chain (270). Phase I of the CTX120 study (NCT04244656) is evaluating the efficacy and safety of anti-BCMA-engineered T cells in patients with relapsed or refractory multiple myeloma (271). An open-label phase I study called COBALT-RCC (NCT04438083) will assess the efficacy, safety, and pharmacokinetics of CRISPR/Cas9-engineered T cells (CTX130) in patients with advanced, relapsed, or refractory renal cell carcinoma (272). NCT03166878 is a phase I/II study evaluating the efficacy and safety of UCART019 gene-edited allogeneic CD19-targeting CAR T cells in patients with relapsed or refractory CD19^+^ leukemia and lymphoma (273). Allogeneic gene-edited dual-specificity CD22, CD20, or CD19 CAR T cells are undergoing a phase I/II trial (NCT03398967) for treating patients with relapsed or refractory leukemia or lymphoma (274). Allogeneic TT52CAR19 T cells (NCT04557436), modified by CRISPR, are being studied in an open-label trial to treat relapsed or refractory CD19^+^ B-cell acute lymphoblastic leukemia in children (275). Trials utilizing CRISPR/Cas9-mediated CCR5 deletion of hematopoietic stem cells in HIV-1 and acute lymphoblastic leukemia patients have been partially successful (NCT03164137), which emphasizes a need for more efficient disruption of CCR5 in lymphocytes (276).

## 4 Regulatory landscape for nanodrug

Nanopharmaceutical development from the manufacturing to scale-up provisions may benefit from the 5R concept, which involves “right target/efficacy”, “right tissue/exposure”, “right patients”, and “right safety”, as proposed (277). Conventional drugs modified into nanoscales for targeted delivery can also be modified in terms of their pharmacokinetics, biodistribution, and toxicokinetic properties; as a result, they foster concerns over quality, safety, and efficacy (278). A number of regulatory authorities worldwide have developed guidelines/frameworks for nanopharmaceuticals in an attempt to ensure transparent, consistent, and predictable regulatory pathways considering safety as well as toxicity (279). Regulatory agencies in their respective jurisdictions include the US FDA, European Medicines Agency (EMA), and Central Drugs Standard Control Organisation, India (CDSCO) (278). The agencies have established guidelines for clinical trials, dossier submissions, and pharmacovigilance as a means of protecting public health (280). Participation by the US FDA is envisioned to establish a science-based approach to the regulation of nanomaterial-based products, build regulatory science knowledge, and facilitate the practice of nanomaterials in regulatory agencies (281). A number of nanodrugs have been developed and approved by the US FDA in collaboration with the National Nanotechnology Initiative (NNI) and Nanotechnology Characterization Laboratory (NCL), which may advance effectiveness and safety measures (281, 282). The EMA is also working to develop regulatory guidelines for the evaluation of nanomedicine products with the European Technology Platform on Nanomedicine (ETPN) and the European Nanomedicine Characterisation Laboratory (EU-NCL) (283). Nanotechnology products are regulated by the FDA and EMA as part of the Innovation Task Force (ITF), an international, multidisciplinary group that includes precise, regulatory, and legal expertise (283). India’s national regulatory authority oversees drug approvals and post-marketing surveillance through the CDSCO, an agency under the Ministry of Health & Family Welfare (284). Nanodrugs approved by the FDA for cancer treatment have different targets including protein synthesis, DNA damage, immunostimulation, microtubule, and hormone inhibition (284). The approved drugs include lipid-based nanoformulation metallic nanoparticles, polymer–drug conjugate drug-targeted antibodies, recombinant viruses, and herbal nanoparticles (285). The FDA- or EMA-approved drugs are DaunoXome^®^, Marqibo^®^, Doxil^®^, Aurimmune^®^, AuNPs^®^, Eligard^®^, SMANCS, Kadcyla^®^, Ontak ^®^, Gendicine^®^, Abraxane^®^, and nanoformulated curcumin (278, 284, 286). An overview of some FDA-approved nanodrugs is depicted in **Table 2**.

**Table 2:** An overview of different types of nanocarriers targeting CSC-specific markers/pathways and the characteristics of their shapes, sizes, and loading abilities, along with applications, advantages, and limitations of nanotherapeutic strategies.

Type of NPs	Size	Structure	Entrapment efficiency (%)	Drug loading (%)	Advantages	Limitations	Applications	References
PLGA NPs	70–200 nm	Shapes may vary, depending on the synthesis methods	27.71 ± 6.86 to 45.70 ± 1.06	8.39 ± 2.1 to 10.62 ± 3.48	Low toxicity, high drug loading capacity, modifiability, high bioavailability, plasticity	Poor drug loading, high immunogenicity, protein adsorption, high burst release, tissue reactions at the site of application, high cost of production, long degradation time, premature drug release, during the preparation of PLGA NPs, asymmetrical distribution of particle size may occur and purification steps are lengthy	Photodynamic and photothermal therapy (PTT), gene therapy, ultrasound-triggered cancer therapy, cancer immunotherapy, combinatorial therapy	(287) (288) (289) (290) (291)
PLGA-PEG colpolymer NPs	100–500 nm	These NPs have PEG shells and core of PLGA for better encapsulation of hydrophilic and hydrophobic drugs	55.2 ± 8.7	9.2 ± 2.23	Better bioavailability, high stability, improved surface hydrophilicity, enhanced circulation time, good biocompatibility, non-toxic	High production cost, accumulation in the body leading to toxicity, the efficiency of PLGA-PEG NPs lacks uniform guidelines for its synthesis and formulation	chemotherapeutic cancer treatments, active targeted cancer therapy, gene-targeting cancer therapy	(190, 248, 254) (292) (293)
Hyaluronic acid NPs	200–450 nm		84.95 ± 1.17	7.38 ± 0.17	Low toxicity, high hydrophilicity, better biocompatibility, modification flexibility, biodegradability, chemical versatility	Low penetration, toxicity, elicit immune system activation, drug resistance, and chemical modification in the structure of HA affect the targeting of CD44 and HA degradation, resulting in unwanted drug release and cellular uptake	Drug delivery systems, tissue engineering, photothermal therapy, photodynamic therapy, chemotherapy, gene delivery, immunotherapy, combination therapy	(294) (295) (296)
Liposomes	50–450 nm	Spherical NPs	87 ± 3.11	4.35 ± 0.15	Biodegradability, low toxicity, high biocompatibility, low immunogenicity, drug hydrolysis resistance, improved biological half-life, amphipathic nature	Storage requirements, poor stability, aggregation of liposomes, toxicity, liposomal phospholipids may undergo oxidation reactions or hydrolysis. Unwanted drug entrapment, reduced solubility, lower bioactivity	Chemotherapy delivery, nucleic acid delivery, cancer immunotherapy, photothermal and photodynamic therapy, stroma remodeling therapy, targeted cancer therapy	(297) (298) (293) (299) (300) (301)
Gold NPs	1–150 nm	Spherical or rod shape NPs	74.57 ± 0.14	16.32 ± 0.023	Good biocompatibility, improved membrane permeability, lower toxicity, hydrophilicity, non-immunogenic	Toxicity of gold NPs and mutagenic effects, accumulation of gold NPs in the spleen and liver and potential effects on kidneys	targeted drug delivery, drug and nucleic acid delivery, photodynamic therapy, X-ray computed tomography (CT) imaging, photothermal therapy	(302) (303) (304) (305)
Micelles	20–80 nm	Core-shell nanostructure, Spherical	53.4 ± 1.15 to 78.7 ± 1.65	5.05 ± 1.01	Low immunogenicity, good biocompatibility, better biodegradability	Poor selectivity, low stability, poor drug loading efficiency, smaller sizes could limit the dose amount	Photothermal therapy, active and passive targeting, photodynamic therapy	(306) (307) (308) (300)
Polymeric NPs	10–1000 nm	Spherical	79.19 ± 0.16	7.19 ± 0.01	High stability, biodegradability, non-immunogenicity, water solubility, biodegradability, encapsulation of both hydrophilic and hydrophobic drugs	Limited targeting ability, non-specific leakage, poor stability, difficulty in scale-up production, alteration in physio-chemical characteristics of NPs, multidrug resistance and early release of drugs, toxicity of polymeric NPs developed due to novel compounds	Drug targeting, passive and active targeting	(309) (310) (311) (312) (313)
Dendrimers	1–10 nm	Polymeric structures, spherical, multibranched, macromolecular, 3D, and multivalent. made up of a core, dendrons, and functional groups at the end	77.8 ± 0.69	6.2 ± 0.06	Better biocompatibility, high drug-loading capacity, improved stability, lower immunogenicity, improved drug half-life	Cationic dendrimers result in maximum toxicity, ROS generation leads to cell death, low solubility of radical dendrimers, high scale-up production, toxicity issues, stability, purification issues, low yield, membrane disruption or erosion upon interaction with bio-membranes	Chemotherapeutic drug delivery, passive targeting, receptor-targeted drug delivery	(314) (315) (316) (317)
Nanodiamonds	2–8 nm	Crystalline structures like diamonds with octahedral conformation	87.8	2.2	Improved tissue penetration and accumulation, enhanced antitumor efficiency, lower therapeutic chemoresistance, lower host toxicity, superior biocompatibility, improved water solubility, lower production cost, fluorescent properties for bioimaging, auto-aggregation, high surface area, and modifiable surface chemistry	Persistent toxicity, accumulation in tissue or organs, modifications, and coatings trigger immune response, especially inflammation, immune system activation, lack of methods that can determine the post-administration distribution inside the body	Targeted drug delivery, active or passive targeting, combined therapeutics co-delivery, targeted gene silencing, diagnostic imaging and labeling, gene delivery, photothermal and photodynamic therapy, marker-based cancer diagnosis, localized chemo-therapeutic elution	(318) (319) (320)
Quantum dots	2–10 nm	Spherical or disc-shaped, made up of core, shell, and surface coating (sometimes)	81.75	4.7	Large surface area to volume ratio, water solubility, photoluminescence, biocompatibility, high photostability, high fluorescence, broad absorption spectra	Difficult size control of QDs, reduction in optical properties due to polymer shell, aggregation may disrupt normal cell function, conjugation increases the QD size leading to reduced delivery to cells, poor biocompatibility, toxicity concerns	Tumor diagnosis, cell imaging, photoinduced therapy, drug delivery	(321) (322) (323) (324) (325)
Carbon nanotubes	10–200 nm	Tubular or fiber-like structure	96.13	44	Biocompatible, water-soluble, chemical stability, large surface area, multifunctional, high conductivity, enhanced flexibility, temperature resistant	Long-term cytotoxicity, deviations in loading of drug–CNT complexes, lack of size uniformity, high cost of synthesis, toxicity concerns	Electrochemical sensing, immunosensing, photoacoustic imaging, fluorescence imaging, drug targeting, photothermal therapy, gene therapy, immunotherapy, photodynamic therapy	(326) (327) (328)

CSC, cancer stem cell; NPs, nanoparticles; HA, hyaluronic acid; ROS, reactive oxygen species; QDs, quantum dots; CNT, carbon nanotube.

## 5 Nanocarrier-mediated drug delivery to CSCs

**Figure 3:**
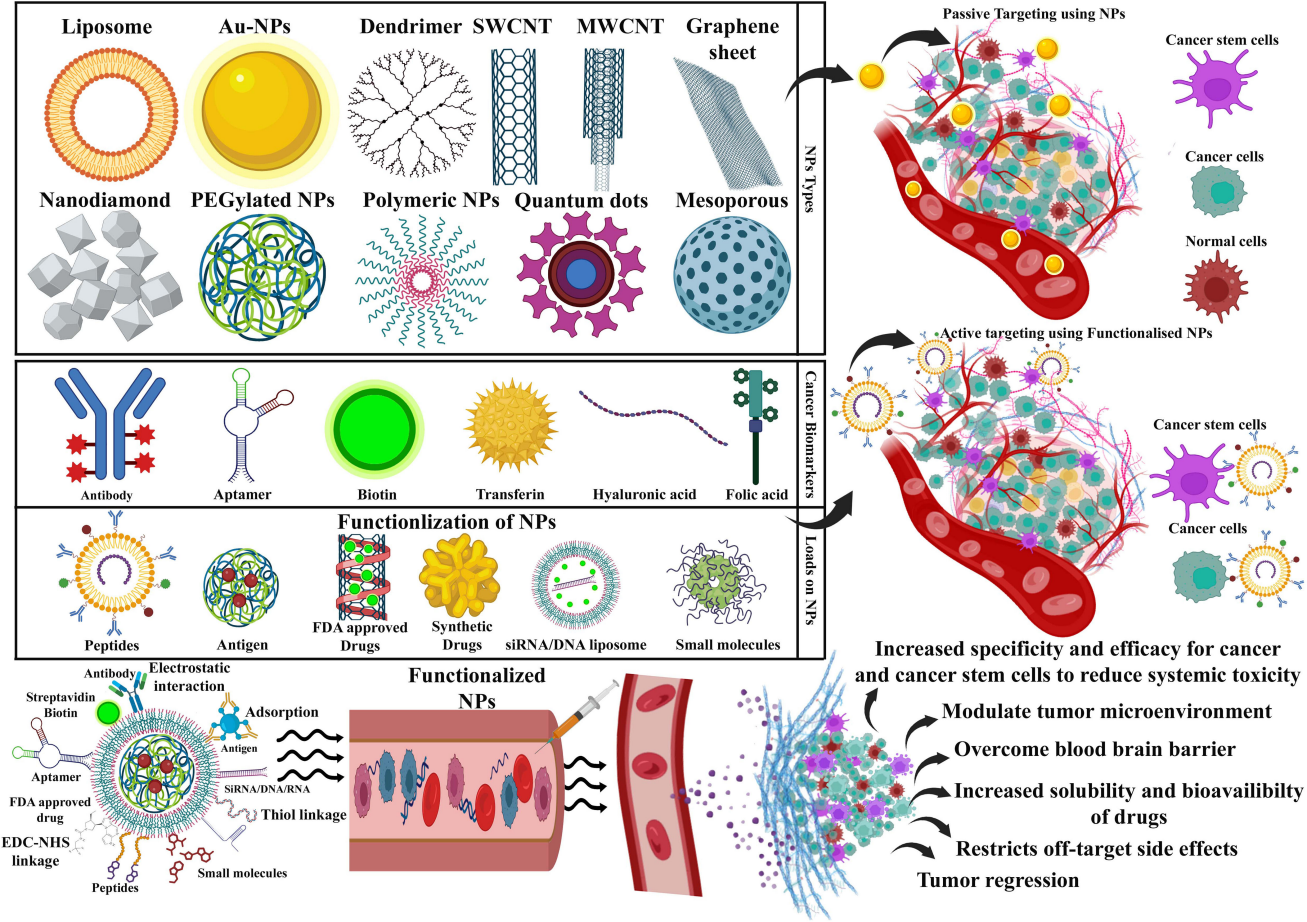
A schematic diagram showing that an array of nanocarriers modified with targeting ligands can, however, specifically attach to tumor cells’ receptors, permitting localized drug delivery or endocytosis. An illustration of active and passive targeting in antitumor nano-delivery systems. Passive targeting is achieved by delivering nanocarriers into tumor tissues via leaky tumor blood vessels, where they accumulate due to enhanced permeability and retention (EPR) effects. Illustration showing the ability of targeted cancer cells to absorb nanocarriers and their accumulation in tumors that exhibit tumor suppression. Image created with BioRender.com.

**Figure 4:**
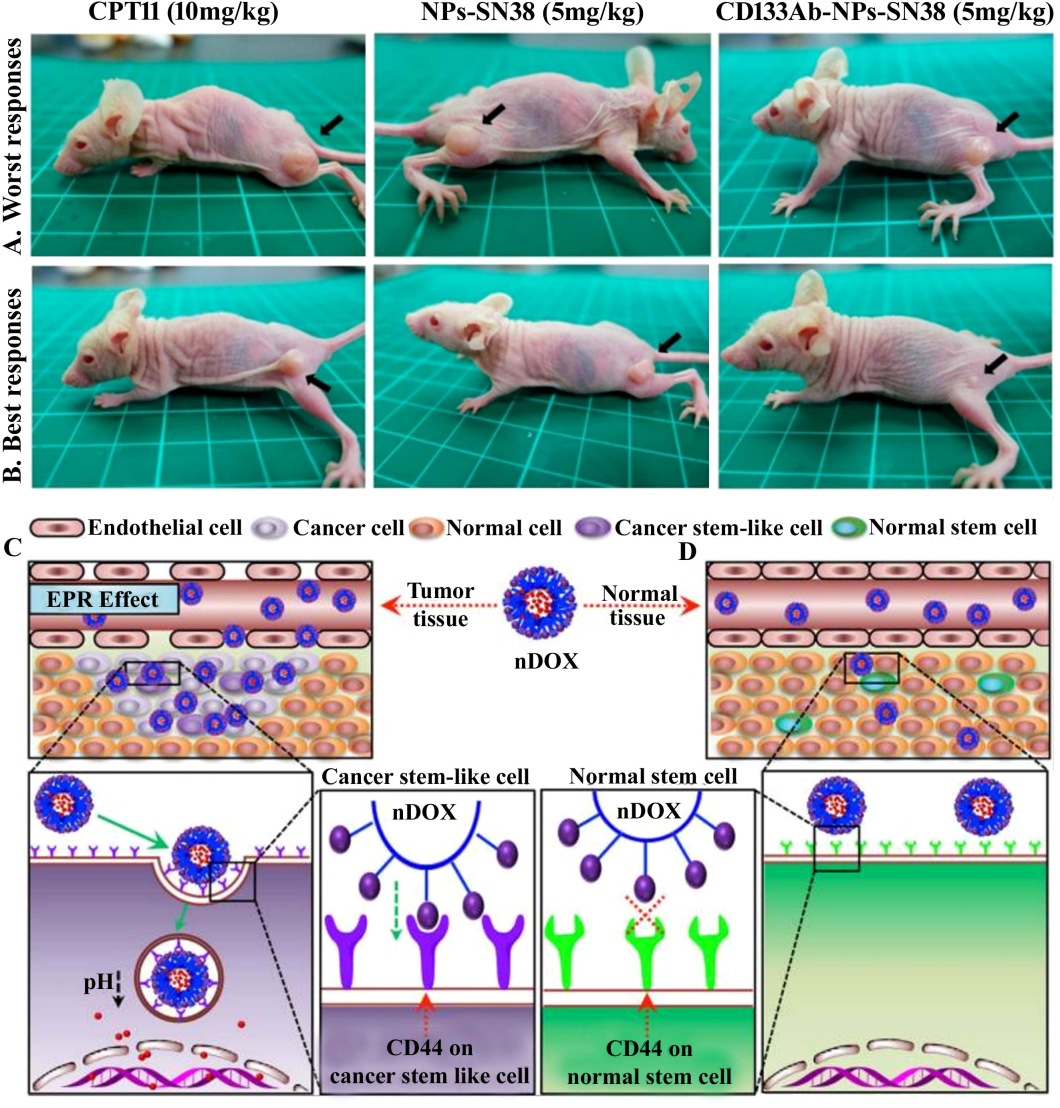
**(A, B)** CD133-positive (CD133^+^) cell was targeted with topoisomerase inhibitor SN-38-loaded nanoparticles conjugated with anti-CD133 antibody to resolve chemotherapy failure. HCT116 overexpress CD133 glycoprotein, which was efficiently bound by anti-CD133 antibody-conjugated SN-38-loaded nanoparticles (CD133Ab-NP-SN-38) demonstrated by the *in vivo* study. The tumor size depiction in mice treated with CPT11 (irinotecan, DNA topoisomerase I inhibitor) as control group, SN-38 nanoparticles (NPs), and CD133Ab-SN38 NPs in HCT116 xenograft model. This CD133Ab-NP-SN-38 combination thwarted tumor growth and hindered recurrence in xenograft model. Reprinted with permission from Ning et al. (381) (Copyright 2016, American Chemical Society). **(C)** CD44-overexpressing breast cancer stem cells (CSCs) were eradicated via chitosan-modified poly(ethylene glycol) (PEG)–poly(propylene glycol) (PPG)–PEG micelle crosslinking loaded with doxorubicin (DOX) in comparison with the free DOX application. DOX-loaded micelles facilitated increased DOX cytotoxicity on cancer stem cell (CSC)-expressing MCF7 breast tumor mouse model exhibiting CD44^+^ overexpression by six times compared to **(D)** normal tissue. Enhanced permeability and retention (EPR) effect of conjugated nanoparticles caused them to accumulate in tumor tissues more than they did in normal tissues. In addition, no noticeable systemic side effects were observed. Reused for illustrative purposes with permission from Rao et al. (382) (^©^ 2015, American Chemical Society).

Nanocarrier used to treat CSCs specifically offers great possibilities. CSC targeting nanocomposite is premeditated based on the notion of ligand–receptor interaction, as tumor tissues express numerous biomarkers distinctively from normal tissues (329–331). The common surface markers between CSCs and normal stem cells protect the latter from the damaging effects of chemotherapeutic agents, despite their similarity in surface markers (10). In addition to enhancing drug accumulation in CSCs, it also protects normal stem cells from therapy-based side effects (332). Compounding the drugs has another advantage in eliminating CSCs due to retrogressive drug resistance, constrained self-renewal, and promotion of differentiation (333, 334). Nanocarriers are colloidal systems with particle sizes below 1,000 nm (335) and were customized in the range of 10 and 200 nm (mainly for drug delivery) (336) to allow the NCs entry into blood vessels within the tumor (123). NCs are modulated with ligands using peptides, antibodies, small molecules, immunotherapeutics, and chemotherapeutics as well as natural polysaccharides that target receptors of CSCs displaying specific binding efficiency and subjected to both preclinical and clinical studies (337) (**Table 3**). A schematic illustration of the ability of ligand-modified NC to target cancer cells is shown in **Figure 3**. A wide range of nano-vehicles mostly enter the cytoplasm over the nucleus where anticancer drugs are highly effective (375). A drug molecule internalizing in the cytosol is unlikely to interact with a subcellular target; therefore, nanoparticle design and optimization are essential to allow cellular/nuclear targeting (376). As a result of leaky tumor vasculatures and poor lymphatic drainage, nanoparticles are more likely to accumulate in tumors than in normal tissues (377). A passive targeting strategy relies on the EPR effect, while active targeting includes targeting tumor cells via ligand-modified nanocarriers that interact with specific receptors (378). Numerous efficient ligands including folic acid, aptamers, hyaluronic acid, biotin, transferrin, peptides, antigens, antibodies, siRNA, small molecules, and FDA-approved drugs (378) have been widely discovered for selective cancer cells and CSCs, reducing localized toxicity, modulating tumor microenvironment, and overcoming blood–brain barrier and drug resistance (**Figure 3**). Kim et al. designed a dual-molecule liposome loaded with doxorubicin and DNA aptamers for the differentiation and targeting of breast tumor cell spheroids and CSCs; one aptamer targets the surface marker mucin 1 antigen (MUC1; a transmembrane glycoprotein) on breast tumor cells, while the other targets the CSC marker glycoprotein CD44 antigen (379). Cho examined CBP4, a small peptide that exhibits an affinity for CD133, a biomarker of glioblastoma cancer stem cells (380) conjugated with gold nanoparticles demonstrating fluorescent signals, was used in glioblastoma imaging and diagnosis. Another study found that topoisomerase inhibitor SN-38 conjugated nanoparticles packed with anti-CD133 antibodies bound efficiently to overexpressing CD133 cells (CD133Ab-NP-SN-38) in HCT116 colon cancer cells (381), showing cytotoxicity and inhibiting colony formation when compared with non-targeted nanoparticles (NP-SN-38) (381). An *in vivo* study in HCT116 xenograft nude mice (**Figures 4A–D**) demonstrated that CD133Ab-NP-SN-38 inhibited tumor growth and delayed tumor recurrence (381). Researchers have developed a polymeric chitosan-coated nanoparticle encapsulated with doxorubicin capable of binding specifically to CD44 receptors, thereby eliminating CD44^+^ cancer stem-like cells and reducing tumor size and cytotoxicity without causing systemic toxicity (382).

**Table 3:** Clinical trials involving nanodrug-based products approved by the FDA and EMA.

Name	Material description	Effective against	Advantages	References
Onivyde^®^ (Merrimack)	Liposomal irinotecan	Pancreatic cancer	Effective delivery to tumor spot; minor systemic toxicity due to side effects	(338, 339)
DepoCyt^©^ (Sigma–Tau)	Liposomal cytarabine	Lymphomatous meningitis	Effective delivery to tumor spot; minor systemic toxicity due to side effects	(340)
Doxil^®^	PEGylated STEALTH^®^ liposomes composed of MPEG-DSPE, HSPC, and CHO	Karposi’s sarcoma	Effective delivery to tumor spot; minor systemic toxicity due to side effects	(341, 342)
Caelyx™	PEGylated liposomal doxorubicin composed of MPEG-DSPE, HSPC, CHO	Kaposi’s sarcoma, multiple myeloma, ovarian and breast cancer, head and neck cancer	Longer circulation time and better off-target profiles	(341, 343)
Marqibo^®^ (Onco TCS)	Liposomal vincristine	Acute lymphoblastic leukemia	Effective delivery to tumor spot; minor systemic toxicity due to side effects	(339)
Myocet^®^ (190 nm)	Liposome-encapsulated doxorubicin citrate	Metastatic breast cancer	Effective delivery to tumor spot; minor systemic toxicity due to side effects	(341)
DaunoXome^®^ (Galen)	Daunorubicin citrate encapsulated in non-PEGylated liposomes composed of DSPC and CHO (2:1)	Karposi’s sarcoma	Longer circulation time, enhanced tumor uptake	(283)
Ontak^®^ (Eisai Inc.)	Engineered protein combining IL-2 and diphtheria toxin	Pancreatic cancer, cutaneous T-cell lymphoma	Lysosomal escape; specificity of targeting T cells	(339) (344)
Abraxane^®^/ABI-007 (Celgene)	Albumin-bound paclitaxel nanoparticles	Ovarian cancer; multiple myeloma, breast cancer, NSCLC, pancreatic cancer	Improved solubility; improved delivery to tumor site	(341) (339)
	Liposome	Prostate cancer		
	Protein nanoparticles	Kaposi’s sarcoma; ovarian cancer; metastatic breast cancer		
Eligard (Tolmar)	Liposome Leuprolide acetate and polymer	ALL	Longer circulation time, precise drug delivery	(283)
Lipo-Dox	Liposome liposomal doxorubicin	Malignant hypothermia	Reduced systemic toxicity of free drug	(341)
Oncaspar (Baxalta U.S.)	Liposome pegaspargase	ALL	Better protein stability, selective targeting of leukemic cells	(345)
Ryanodex (Eagle Pharmaceuticals)	Liposome dantrolene sodium	Breast cancer, pancreatic adenocarcinoma	Higher dose administration at a faster rate	(346)
EndoTAG-1 (SynCore Biotechnology)	Lipid-based nanoparticle with paclitaxel	Non-small-cell lung cancer, breast cancer	Cancer cytotoxicity and cytostatic potency	(347) (348)
Tecemotide (Merck KGaA)	Lipid-based nanoparticles with MUC1 antigen	Advanced solid tumors, lung, biliary, bladder, or pancreatic cancers	sMUC1 or ANA biomarkers elevated	(349)
MM-302 (Merrimack Pharmaceuticals)	Lipid-based nanoparticles with doxorubicin hydrochloride	Solid tumor malignancies	MM-302 failed to validate benefits over the control, hence not approved	(350)
Nanoplatin (NanoCarrier)	Polymer-based nanoparticles with cisplatin	Renal and rectal cancer, ovarian cancer	Longer blood circulation, enhanced accumulation in tumor tissues, tumor growth inhibition	(351)
CT-2106	Polymer-based poly(L-glutamic acid) nanoparticles	Melanoma	Longer plasma half-life, less renal clearance, and good solubility	(352)
CRLX101 (Cerulean)	Polymer-based nanoparticles with cyclodextrin-camptothecin	Melanoma	Increased distribution to tumor tissues with good tolerability	(352–354)
Taxoprexin (Luitpold Pharmaceuticals)	Lipid-based nanoparticles with paclitaxel	Breast and brain metastases	Less toxicity and enhanced tumor response	(355)
Allovectin-7^®^ (Vical)	VCL-1005 plasmid	Melanoma	Effective for melanoma stage III/IV	(356)
NKTR-102 (Nektar Therapeutics)	Polymer-based nanoparticles with irinotecan	Pancreatic cancer	Significant advance in patient’s survival rate	(357)
MAGE-A3+AS15 (GSK)	Lipid-based nanoparticles with human melanoma-associated antigen A3 protein	Esophageal and gastroesophageal junction adenocarcinoma	Market withdrawal of MAGE-A3 immunotherapeutic	(358)
NC-6004 (NanoCarrier)	Polymer-based nanoparticles with cisplatin	Non-small-cell lung tumor, breast tumor, gastric tumor	Effective antitumor potency	(359)
SP1049C	Polymeric micelle with Pluronics^®^ L61/F127	Sarcoma, advanced lung and liver metastases hepatocellular carcinoma, head and neck squamous cell carcinoma	Multi-pathway pro-apoptotic inhibition	(360
Lipoplatin	Polymeric micelle PEGylated cisplatin liposomal platinum drug formulations	Breast cancer	Benefits chemotherapy-resistant patients	(352)
NBTXR3 (Nanobiotix)	Inorganic nanoparticles with hafnium-oxide nanoparticle	Breast, colorectal, and lung tumor	Boosts tumor efficacy, reducing toxic effects on healthy tissue, non-surgical endoscopic drug delivery for pancreatic cancer patients	(361, 362)
NK-105 (NanoCarrier)	Polymer-based nanoparticles with paclitaxel	Metastases in the brain	Effective tumor size reductions, progression-free survival was not achieved	(363–365)
FCE28068/PK1	Polymer–drug conjugate with DOX–polymer conjugate	Ovarian, fallopian tube, or peritoneal cancer	Efficacious against cancer with minimal side effects	(366)
2B3-101	Liposome glutathione PEGylated liposome with doxorubicin hydrochloride	Liver and breast tumor	Prevents tumor growth and survival prolongation	(367)
XYOTAX CT-2103 (CTI BioPharma)	Poly(l-glutamic acid) with paclitaxel	Primary or metastatic liver cancer	Increased tumor eradication efficacy while reducing toxicity to healthy tissues	(368)
Thermodox^®^	Polymer–drug conjugate PEG with doxorubicin	Breast tumor, ovarian tumor, pancreatic tumor, non-small cell lung tumor	Effective treatment 25 times greater than doxorubicin (unapproved)	(352)
FCE28069/PK2	Polymer–drug conjugate galactosamine-*N*-(2-hydroxypropyl)methacrylamide doxorubicin	Metastatic pancreatic cancer	Asialoglycoprotein receptor-mediated active targeting of liver cancer	(369)
Genexol-PM	Polymeric micelle PEG-P(d,l-lactide) with paclitaxel/carboplatin/gemcitabine	Acute myeloid leukemia	MTD increased by threefold, without triggering hypersensitivity	(352)
NK911	Polymeric micelle doxorubicin-conjugated poly-aspartic acid/polyethylene glycol	Breast cancer	EPR effect causes antitumor activity	(352)
CPX-351 (Vyxeos™)	Liposome daunorubicin+cytarabine	Ovarian tumor, advanced non-small-cell lung tumor, DOX-resistant breast cancer	Well tolerated and leads to morphologic remission	(370) (371)
EndoTAG^®^	Liposome with paclitaxel (DOTAP, DOPC, PTX (50:47:3)	Epithelial ovarian carcinoma	Phase II trial showed good efficacy and survival in triple-negative breast cancer and advanced pancreatic cancer	(370) (371)
SPI-77	Liposome PEGylated liposomal formulation of cisplatin	Gastric, ovarian, and lung cancer, non-small-cell lung cancer	Phase III clinical trials were not conducted due to inactive antitumor activity	(352)
PTX–LDE (paclitaxel-lipid core nanoparticle)	Liposome with paclitaxel (135 mg cholesteryl oleate, 333 mg egg PC, 132 mg Miglyol 812 N, 60 mg PTX, 6 mg cholesterol)	Solid tumors and hematological malignancies	Tumor arrest with minimal side effects	(370, 372)
Lipusu^®^	Liposome with paclitaxel (72 g PC, 10.8 cholesterol in ethanol)	Gastric and ovarian malignancies, non-small-cell lung cancer	Antitumor efficacy with lower toxicity on bone marrow, lower cardiotoxicity	(371, 373)
MRX34	Liposome with miR-34a (DOTAP, cholesterol)	Refractory advanced solid tumors	Subset of patients with resistant solid tumors showed antitumor activity	(371, 374)

DSPC, distearoyl phosphatidylcholine; DOPC, dioleoyl phosphatidylcholine; DOTAP, dioleoyl trimethylammonium-propane; PTX, paclitaxel; PC, phosphatidylcholine, poly(ethylene glycol)-*b*-poly(d,l-lactide); MTD, maximum tolerable dose; EPR, enhanced permeability and retention; miRNA-34, microRNA-34; MAGE A3, melanoma antigen family A3; DOX, doxorubicin; MUC1, mucin 1; ANA, antinuclear antibodies; ALL, lymphoblastic leukemia; NSCLC, non-small-cell lung cancer; IL-2, interleukin-2; CHO, cholesterol; HSPC, fully hydrogenated phosphatidylcholine; MPEG-DSPE, *N-*(carbonyl-methoxy(polyethylene glycol)-2000)-1,2-distearoyl-*sn*-glycero-3-phosphoethanolamine sodium salt; FDA, Food and Drug Administration; EMA, European Medicines Agency.

### 5.1 PLGA NCs

Poly(lactic-co-glycolic acid) (PLGA) is widely employed for the preparation of drug-loaded NCs due to its biodegradable properties and several applications in biomedical compounds (155). PLGA NCs are employed as a paclitaxel carrier in the case of ovarian cancer stem cells (378, 383). PEGylated poly(lactic-co-glycolic acid) carriers containing salinomycin (SAL-NP) and CD133 aptamers (Ap-SAL-NP) efficiently stopped the progression of CD133^+^ osteosarcoma cancer stem cells (384). Jin et al. demonstrated that GE11 peptides conjugated with PLGA NCs can deliver the conjugated anticancer agent, curcumin, to cells expressing EGFR receptor (EGFR) *in vitro* and *in vivo* (385). When these curcumin-loaded NCs were applied to breast cancer cells and tumor-bearing mice, the signaling of phosphoinositide 3-kinase was reduced, cancer cell viability was diminished, drug clearance from the bloodstream was attenuated, and tumor growth was reduced (385). After being delivered in the form of GE11-Cur-NPs, Cur rapidly accumulates within MCF-7 cells, suggesting active receptor-mediated endocytosis as well as passive uptake through the cell membrane (385). Pancreatic CSCs are inhibited by anthothecol-encapsulated PLGA NCs (Antho-NCs) through the inhibition of the sonic hedgehog pathway (386). Antho-NCs established show a therapeutic role demonstrated by reduced cell motility, migration, and invasion by upregulating E-cadherin and obstructing N-cadherin and Zeb1 (386). The antagonistic effect of Antho-NCs on pluripotency-maintaining factors and stem cell markers indicates that they are blocking CSC generation, disrupting Gli binding to DNA, and inhibiting Gli transcription (386). The dual inhibition of AKT and mTOR by nimbolide-loaded PLGA nanocarriers induces mesenchymal-to-epithelial transition in pancreatic cancer stem cells (387).

### 5.2 PLGA-PEG copolymer NCs

PLGA-PEG has been employed for the simultaneous delivery of various chemotherapeutic drugs for colorectal cancer therapy and lung cancer treatment (254). Dhar et al. (388) created prostate-specific membrane antigen (PSMA) targeting NCs using Pt(IV)-encapsulated PLGA–poly(ethylene glycol) (PEG)-functionalized controlled-release polymers for targeting cisplatin delivery to prostate CSCs (388). FA-modified NCs encapsulating CDDP and paclitaxel (PTX) exhibited superior targeting and antitumor efficacy against M109 cells (389). Cisplatin-encapsulating maleimide-polyethylene glycol-poly(d,l-lactic-*co*-glycolide) (mal-PEG-PLGA) in synergy with a CD44 monoclonal antibody produced via electrospray technique was effective at inhibiting ovarian cancer cell proliferation compared with cisplatin in free form and PLGA without CD44-conjugated NPs (390). Core-shell NCs fabricated using double emulsification of an amphiphilic copolymer, methoxy poly(ethylene glycol)-poly(lactide-*co*-glycolide) (mPEG-PLGA), were employed for simultaneous delivery with hydrophilic doxorubicin (DOX) and hydrophobic paclitaxel (TAX) (391). Despite the same concentrations of DOX and TAX, NCs suppressed tumor cell growth more efficiently than both on their own in A549, B16, and HepG2 cells (391). The authors suggested that DOX intercalates DNA, thereby interfering with transcription, which interrupts tubulin synthesis. The treatment also degrades microtubules, subsequently reducing microtubule content in tumor cells (391).

### 5.3 PLA-PEG NCs

Polylactic acid (PLA) is a biodegradable polymer as declared by the FDA, and it is found to be completely excreted through metabolism. Fabricated docetaxel (DTX) PLA NCs targeting lung cancer stem-like cells (CSLCs), on administration, indicated observable inhibition in tumor growth and anti-metastatic efficacy (392). Studies have shown that encapsulating salinomycin (SAL) in PLA NCs improved its pharmacokinetics and biodistribution profile, demonstrating efficacy against chemo-resistant cancer cells and CSCs (393). Also, when administered to Ehrlich ascites carcinoma (EAC) tumor-bearing mice, SAL: DOX co-loaded NCs caused significant tumor regression and complete inhibition of cancer recurrence (393, 394). Ahmadi-Nouraldinvand and colleagues designed PLA-PEG-based NCs, namely, PLA-chitosan-PEG-folic acid (COPA), PLA-chitosan-PEG-glucose (COPB), COPA and COPB (COPAB), and chitosan-PLA-PEG-FA/Glu/VEGF/siRNA/PTX (NCsAB/siRNA/paclitaxel for efficient siRNA and paclitaxel drug delivery to MCF-7 cells (395). The author opined that the release of siRNA and paclitaxel nanocarrier was favorable due to the acidic environment of tumor tissues (395). Curcumin and bortezomib, both slightly water-soluble anticancer drugs, were loaded as a complex (curc-BTZ) into methoxy-poly(ethylene glycol)-block-polylactic acid (mPEG-*b*-PLA) diblock copolymers, which demonstrated induced cytotoxicity in HeLa, MCF-7, and MDA-MB 231 cells (396).

### 5.4 Hyaluronic acid NCs

HA is an anionic, non-sulfated glycosaminoglycan that exhibits biocompatibility, biodegradability, and non-immunogenic properties, making it an excellent candidate for conjugating different drugs in cancer treatment (397). HA-functionalized NCs co-delivering camptothecin (CPT) and curcumin (CUR) (HA-CPT/CUR-NCs) exhibit synergistic anticancer effects, making HA-CPT/CUR-NCs a promising approach for colon cancer-targeted therapy (398). Inhibitory effects of naproxen nanoparticles coated with hyaluronic acid (HA) are demonstrated in breast cancer stem cells through modifications in the GSK-3β-related COX-independent pathway, providing a controlled release of naproxen, leading to apoptosis (399). An effective binding of HA (HA-eNCs) to CD44-enriched B16F10 cells was observed when all-*trans*-retinoic acid (ATRA)-encapsulated cationic albumin functionalized with HA (HA-eNCs) was applied to CSCs overexpressing CD44, triggering targeted delivery of drugs to eradicate CSCs (400).

### 5.5 Liposomes

Liposomes are characterized by self-accumulated vesicles consisting of a bilayer of lipids that completely encircles an internal aqueous phase (401). Liposomes can be a drug carrier to both hydrophilic and hydrophobic molecules, which is its major advantage (402). Using anti-CD44 antibodies, Wang et al. delivered liposomal NCs loaded with Dox and triple fusion (TF) genes consisting of the herpes simplex virus truncated thymidine kinase (HSV-ttk), renilla luciferase (Rluc), and red fluorescent protein (RFP) (403). As a result, non-invasive molecular imaging techniques were developed for monitoring and evaluating targeting efficacy and gene therapy in hepatocellular carcinoma (HCC) cells (403). CD133^+^ glioma stem cells undergo selective apoptosis and differentiate into non-stem-cell lineages following administration of dual-modified cationic liposomes (DP-CLP) with survivin siRNA and paclitaxel (404).

### 5.6 Gold nanocarriers

Gold nanocarriers (AuNCs) are known to lack the ability to induce adverse and acute toxicity; due to their unique optical properties, remarkable biocompatibility, easy turning of physicochemical properties, and surface chemistry (405, 406), they have been considered as a potential contrast agent in *in vivo* imaging (407). AuNCs conjugated with the antimetabolite 5-fluorouracil (5-FU) and CD133 antibody could enhance specific targeting by AuNPs and therefore reduce non-specific binding, thus reducing the possibility of systemic side effects in colorectal cancer CSCs (408). Gold nanoparticles were modified by modifying their surfaces with 6-mercapto-1-hexanol so that protoporphyrin IX and folic acid could be conjugated simultaneously for improved internalization through photochemical processes (409). The results showed that when compared to conventional photodynamic therapy, selective phototoxicity was increased in cancer cells (409). A combination of 5-aminolevulinic acid (5-ALA)-bound AuNCs and photodynamic therapy (PDT) decreased the invasion of cutaneous squamous cell carcinoma cells and the migration potential of the cells (410).

### 5.7 Micelles

Self-assembling nanomicelles (10–100 nm) are colloidal dispersions with a hydrophobic core and a hydrophilic shell (411). Boosted tumor suppression and apoptosis *in vivo* were observed in H460 human lung cancer cells and CSCs with the application of phenformin-loaded micelles (Phen M) along with gemcitabine-loaded micelles (Gem M) (412). The development of poly(styrene-*b*-ethylene oxide) (PS-*b*-PEO) and poly(lactic-*co*-glycolic) acid (PLGA) by double emulsions loaded with covalently bound temozolomide (TMZ) and/or RG7388 (idasanutlin) to CD133 aptamer, resulting in the possibility of targeting glioblastoma CSCs in combination with simultaneous diagnostic imaging, has been demonstrated (413). Ghosh and Biswas developed Pluronic P105 micelles loaded with doxorubicin and PTX loaded with dextran stearate were used to target melanoma folate-positive B16F10 cells and breast cancer cells (414).

### 5.8 Polymeric NCs

Polymeric NCs (PNCs) are hydrophilic cores that are surrounded by a polymeric substance with a size range of 1–1,000– nm used by Sun et al. to target gastrointestinal CSCs (415). NanoCurcTM, a polymer-encapsulated curcumin nanoparticle formulation, significantly enhanced brain CSC treatment by augmenting curcumin’s bioavailability and encouraging apoptosis, cell cycle arrest, growth reductions, and clonogenicity in brain CSCs with a reduction in CD133^+^ population of brain tumors (58). A study demonstrated efficient delivery of salinomycin to the EGFR−overexpressing osteosarcoma CSCs and cancer cells, which led to a reduced CSC population on osteosarcoma cells and CSCs by EGFR aptamer-bound, salinomycin-loaded polymer-lipid hybrid nanocarriers (EGFR-SNCs) (416).

### 5.9 Dendrimers

A dendrimer is defined as a three-dimensional macromolecule with multiple polymeric branching having the capability of structural modifications (417). Dendrimers are studied for their application in drug and gene delivery including poly(propylene imine) (PPI), poly−l−lysine (PLL), polyamidoamine (PAMAM), polyglycerol, poly(etherhydroxylamine) (PEHAM), and poly(ester amine) (PEA) (418). A temozolomide-loaded polyamide-amine dendrimer in a PAMAM delivery system was developed to explore its potential in targeting melanoma cells *in vitro* (419). Li et al. targeted CD44^+^ gastric cancer cells with hyaluronic acid-modified polyamidoamine dendrimer G5-entrapped gold NCs bound to the METase gene, resulting in repressed tumor growth of gastric cells (420). The study of Kesharwani et al. (421) utilized a CD44-targeted G4 PAMAM dendrimer combined with HA, followed by 3,4-difluorobenzylidene curcumin (CDF) for targeting MiaPaCa-2 and AsPC-1 cells. HA-PAMAM-CDF increased the cytotoxicity and antitumor activity in MiaPaCa-2 cells compared to AsPC-1 cells (421).

### 5.10 Quantum dots

Quantum dots (QDs) are semiconductor nanocarriers with excellent fluorescence properties and have been shown to possess significant imaging, sensing, and therapeutic advantages for cancer treatment in its earliest stages (422). QDs conjugated to anti-HER2 were used for immunolabeled breast and lung cancer cells and showed superior performance in a panel of lung cancer cells with differential HER2 expression, suggesting that they may be a useful tool for the identification of cancer biomarkers at an early stage (423). A study of QDs using EGFR mutation-specific antibodies showed superior effectiveness and sensitivity to traditional mainstays in determining patients’ disease status and therapeutic decisions (424). Researchers have demonstrated the performance of QD-based miRNA nanosensors for detecting point mutations in mir-1962a2, which is abnormally expressed in NSCLC patients’ lung tissues (425). To study the cytotoxicity pathway in hepatocellular carcinoma HepG2 cells, Nguyen et al. (426) synthesized a cadmium telluride quantum dot (CdTe-QD) method, which exhibited apoptosis in HepG2 cells following improved caspase-3 activity, poly ADP-ribose polymerase (PARP) cleavage, and phosphatidylserine externalization. Moreover, augmented activity of Fas levels and caspase-8 markers for extrinsic apoptosis pathway were also observed due to CdTe-QDs (426).

### 5.11 Nanodiamonds

Nanodiamonds (NDs) possess properties like biocompatibility and efficient drug delivery capability, making them a crucial nanoparticle-based vehicle (427). Nanodiamonds upon cracking form very-small-sized semi-octahedral carbon structures with crystallographic surfaces and sharp edges (428). Their surfaces can be used with small molecules, imaging agents, therapeutic biomolecules, genetic material, and targeting ligands, i.e., by a wide range of biological and chemical agents (429, 430). Chemoresistance was overwhelmed in hepatic cancer cell lines when an epirubicin-nanodiamond complex (EPND) was prepared, exhibiting enhanced efficiency compared with the original epirubicin (431). The dissociation of epirubicin from ND can be trigged by intracellularly charged protein molecules (431). It was found that micropinocytosis is crucial for the uptake of EPND, while inhibitors of clathrin-mediated endocytosis may weaken the uptake of EPND (431).

### 5.12 Carbon nanotubes

Carbon nanotubes consist of crystalline graphene; express exceptional properties like solubility in water, membrane penetration, discrimination of tumor retention, high drug loading capacity, less toxicity, and Raman properties; and are important for nanotechnology and clinical research (432–438). Research has shown that carbon nanotube-mediated thermal treatment can ablate both bulk breast tumors and breast cancer stem cells, impacting tumor growth, proliferation, and recurrence (439). A multimodal single-walled carbon nanotube (SWCNT) functionalized with CD44 antibodies established selective anti-CD44 targeting, providing effective therapy against breast CSCs (440). Distearoylphosphatidylethanolamine–hyaluronic acid (DSPE-HA) nanotubes were synthesized with a single coupling point in order to yield SWCNTs (DSPE-HA SWCNTs) with high dispersion and biocompatibility for targeting CD44-overexpressing cells (441). They developed novel drug delivery systems for epirubicin (EPI) using DSPE-HA SWCNTs as carriers, i.e., EPI-SWCNTsDSPE-HA (441). A549/Taxol cells and tumor spheroids were treated with EPI-SWCNT-DSPE-HA complexes for efficacy testing. It was found that EPI-SWCNT-DSPE-HA significantly increased intracellular EPI accumulation via CD44 receptor-mediated endocytosis in multidrug-resistant cancer cells (441).

## 6 Extracellular vesicles

### 6.1 Cellular exosomes: sources and structures

**Figure 5:**
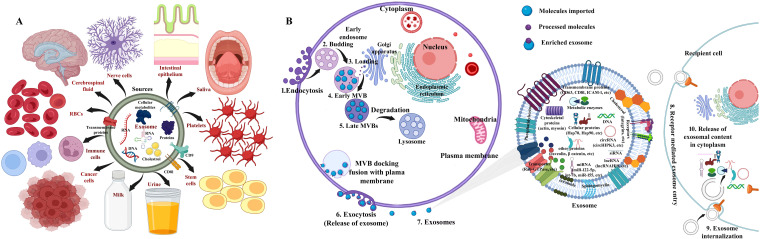
**(A)** Schematic depiction for sources of exosome: stem cells, cancer cells, immune cells, nerve cells, saliva, platelets, red blood cells (RBCs), breast milk, cerebrospinal fluid, intestinal epithelium, and urine, among others. **(B)** The generalized structure of exosomes and overview describing the biogenesis of exosomal stem cells and cargos and consequent intake of exosomes by recipient cells. (1) Fusion with target recipient cell, (2) endocytosis, and (3) interaction with specific receptors on cell surface and internalization. Source: Vakil et al. (442). Image was modified and recreated using BioRender.com.

Extracellular vesicles (EVs) are lipid bilayer NCs found in the cytoplasm with diameters ranging from 30 to 2,000 nm, comprising sugars, nucleic acids, proteins, and lipid biomolecules (382). The smallest EVs ascended from multi-vesicular endosomes. Stem cells, cancer cells, immune cells, nerve cells, and other cell types secrete exosomes, which are found in saliva, amniotic fluid, tears, breast milk, platelets, plasma, red blood cells (RBCs), cerebrospinal fluid, bronchial fluid, synovial fluid, intestinal epithelium, nerve, urine, semen, lymph, bile, and stomach acid, are illustrated in **Figure 5A** (443–445). The plasma membranes of EVs begin budding inward, forming early endosomes that later develop into late endosomes after maturation and multivesicular bodies (MVBs) with intraluminal vesicles (ILVs) (446). Plasma membranes and MVBs fuse to release exosomes; microvesicles are delivered by direct budding of the plasma membrane externally (447). EVs have plenty of cargos, such as proteins, nucleic acids, metabolites, and lipids (448). EVs are uptaken by recipient cells via various processes including endocytosis, ligand–receptor interaction, and direct fusion as shown in **Figure 5B** (442).

### 6.2 Exosomes in antitumor therapy

In recent years, naturally secreted exosome vesicles have attracted significant attention as drug delivery vehicles due to their similarities with liposomes (449). A nanometric exosome is easily transported between cells; a lipid bilayer membrane protects bioactive molecules from degradation in the extracellular environment (449–451). Several advantages of exosomes have been demonstrated, including their ability to combat CSCs, lower immunostimulatory, extensive circulation time, and eminent loading efficacy, making them ideal as nanocarriers for drug loading and/or delivery (452, 453). Cheng and co-workers isolated exosomes from healthy hepatoma cells and transfected them using lentivirus expressing p120ctn; as a result, hepatocellular carcinoma cells formed fewer colonies, decreased proliferation, and inhibited migration (454). Furthermore, the exosomes with p120ctn expression reduced the tumor growth in *in vivo* hepatocellular carcinoma xenograft mice (454). It was also observed that exosome p120ctn did not impact PI3A/Akt or MEK/ERK pathways; however, STAT3 phosphorylation was vividly decreased in hepatocellular carcinoma cells, suggesting that the exosome p120ctn disables STAT3 to impede the hepatocellular carcinoma cell proliferation, metastasis, and expansion of the respective CSCs (454). Hu et al. (360, 455) reported that the exosomes secreted by stromal fibroblasts promote the reversion of phenotype and attainment of CSC characteristics in differentiated colorectal cancer cells by triggering Wnt signaling (360, 455). The *in vitro* and *in vivo* experiments suggested that inhibition of Wnt release using the porcupine inhibitor LGK974 curtailed the drug resistance in differentiated colorectal cells and possibly impacted CSC stemness (360, 455). Interestingly, a recent study determined that the migration and invasion of M2 macrophage-modulated colorectal cancer cells are controlled by M2 macrophage-derived exosomes, expressing higher levels of miR-21-5p and miR-155-5p, which are crucial to exosome-mediated colorectal cancer cell migration and invasion (456). Lin et al. (457) introduced that exosomal miR-21-5p derived from bladder cancer cells reversed phosphatase and tensin homolog instigation of the PI3K/AKT pathway in macrophages; in contrast, it induced STAT3 expression to promote the M2-polarized differentiation of tumor-associated macrophages (457). The secreted exosomal miR-21a-5p from the M2 macrophage induced the differentiation and proliferation of pancreatic cancer stem cells by targeting KLF3 for attenuating the stemness of pancreatic cancer (458). Moreover, downregulation of miR-21a 5p in M2 macrophage-induced EVs reduced the expression of Nanog/Oct4 and reduced sphere formation, colony formation, migration, invasion, and anti-apoptosis potency of pancreatic CSCs both *in vitro* and *in vivo* (458). The authors focused on miR-21-5p mediated KLF3 downregulation and targeted the differentiation ability of pancreatic stem cells (458). Unique miRNA has been found in prostate cancer exosomes resulting from cancerous stem cells and non-cancerous stem cells (459). Moreover, future cancer cell spread environments are prepared using CSC-derived exosomes (460).

**Figure 6:**
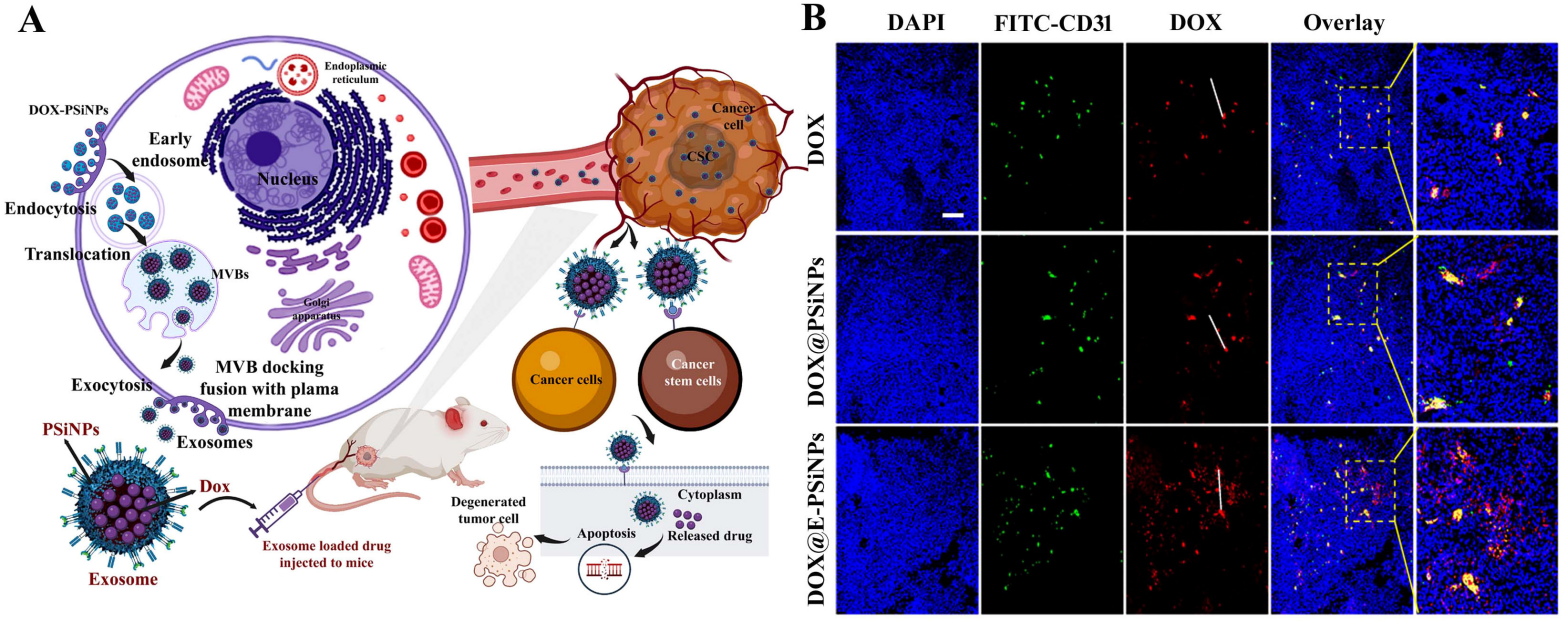
**(A)** The exosome-encapsulated porous silicon nanoparticles (E-PSiNPs) and DOX@E-PSiNP preparation as antitumor drug carriers are shown in schematic diagram. After incubation, DOX@E-PSiNPs are endocytosed into cancer cells, localized to multivesicular bodies (MVBs), and form autophagosomes. Exocytosis of DOX@E-PSiNPs occurs upon fusion of MVBs with cell membranes. A systemic injection of DOX@E-PSiNPs in tumor-bearing mice resulted in strong anticancer activity displaying accumulation in both cancer cells and cancer stem cells (CSCs) and penetrating deeply into tumor tissues. **(B)** Florescent images showing the localization of both DOX and CD31-labeled tumor blood vessels in tumors isolated from H22 tumor-bearing mice at 24 h after intravenous infusion of DOX alone, DOX@PSiNPs, and functional exosome-encapsulated DOX-PSiNPs (DOX@E-PSiNPs) at DOX dosage of 0.5 mg/kg; scale bar, 200 µm. A wide distribution of DOX@E-PSiNPs was evident after treatment; conversely, DOX and DOX@PSiNPs accumulated mostly around blood vessels as evidenced by stronger co-localization with FITC-CD31-labeled endothelial cells. A white line outlines the gap between the DOX distribution in blood vessels and the tumor parenchyma. Reused for illustrative purposes with permission from Yong et al. (467) (Copyright 2019, source: Tumor exosome-based nanoparticles are efficient drug carriers for chemotherapyNature Communications).

The ability of NCs to induce autophagy has been reported for silver nanomaterials and carbon- and silicon-based nanomaterials (461–463). Pfeffer (464) reported the release of exosomes in certain cell types regulated by Rab27A (464) and Rab27B GTPases and their cognate effector proteins. As a next step, Chen et al. (465) investigated the impacts on parental cells subsequently preventing the exosomal release factor by impeding Rab27a-dependent exosome secretion (465). Downregulation of self-created exosome secretion (Rab27a) from metastatic hepatocellular carcinoma (MHCC97H) inhibited the migration, chemotaxis, and invasion of intrahepatic and lung metastasis via the MAPK/ERK signaling pathway, thereby targeting EMT (465). One of the most notable advantages of exosome-mediated doxorubicin delivery is its dramatic reduction in cardiotoxicity, which is commonly associated with doxorubicin in clinical applications (466). Yong et al. developed biocompatible tumor cell exosome-sheathed PSiNPs (E-PSiNPs) that can be exocytosed by tumor cells for targeted cancer chemotherapy (467). DOX was conjugated to luminescent porous silicon nanoparticles (PSiNPs, 150 nm) (DOX@PSiNPs) and incubated with H22 hepatocellular carcinoma tumor resulting in engulfment of exosome-sheathed (DOX@E-PSiNPs) (467). The DOX@E-PSiNPs enable them to enrich *in vivo* in both tumor cells and CSCs, resulting in DOX uptake by CSCs with eventual eradication of the CSCs (467) and facilitating the effectiveness in destroying subcutaneous, orthotopic, and metastatic cancer. The schematic illustration of nanocarrier design and its application in eradicating CSCs is presented in **Figure 6A**. Further, intravenous injections of free DOX, DOX@PSiNPs, and DOX@E-PSiNPs were used to determine whether DOX@E-PSiNPs penetrate deeply into tumors in xenograft mice of H22 hepatocellular carcinoma. The confocal microscopy images showed widespread distribution of DOX@E-PSiNPs in complete tumor sections after 24 h (**Figure 6B**), while DOX@PSiNPs and free DOX were mostly accumulated around the blood vessels as evidenced by FITC-CD31-labeled endothelial cells. A white line delineates the gap between the DOX distribution in blood vessels and the tumor parenchyma (467).

### 6.3 Clinical trials of exosomes in cancer therapy

The European Union, Australia, and the United States have regulatory frameworks for manufacturing and conducting clinical trials, but there may be a need for guidelines dedicated to EV-based therapeutics (468). Exosomes have shown promising results *in vitro* and in animal models, indicating that they can be used to target CSCs; some clinical trials have already achieved significant results (469). Ascite (Aex)-derived exosomes together with granulocyte–macrophage colony-stimulating factor (GM-CSF) have been tested in a phase I clinical trial for the immunotherapy of colorectal cancer (470–472). This combinational immunotherapy shows the induction of beneficial tumor-specific antitumor cytotoxic T lymphocyte response, but not in the case of Aex alone, indicating the feasibility and better tolerance capability of patients with colorectal cancer (473). An intradermal and subcutaneous immunization of stage III/IV melanoma patients with autologous dendritic cell exosomes pulsed with melanoma-associated antigens family (MAGE 3) peptides was shown in a phase I trial (474). Exosome administration in these patients has proven to be safe and feasible, despite neither CD4^+^ nor CD8^+^ T cells specific to MAGE3 being detected in peripheral blood (474, 475). In a phase I study conducted by Morse et al. (476), patients with advanced non-small-cell lung cancer showed improved immune response and tumor progression after receiving dexosome (DC-derived exosomes loaded with the MAGE tumor antigens) immunotherapy. Pulsed dendritic cell EVs activated cytotoxic T cells against a growing tumor in immune-competent mice (477–479). According to Viaud et al. (480), dendritic cell-derived exosomes promote natural killer cell activation and result in anti-metastatic effects, which may be related to NKG2D ligands and IL-15Ralpha. Clinical regressions observed in the first phase I trial using peptide-pulsed Dex (dendritic cell-derived exosomes) were attributed to reduced T-cell response (481, 482). Phase II trials showed that IFN-γ-DC-derived exosomes were capable of boosting antitumor immunity in advanced non-small-cell lung cancer patients following phase I and preclinical trials (475). EVs generated by pulsed DCs, rather than those made by MHC class I and II peptides, induced the activation of B cells and promoted tumors (483, 484). When tumor EVs are combined with appropriate immune-stimulating adjuvants, their immune-inhibitory effect can be suppressed, enabling them to stimulate antitumor responses in advanced ovarian cancer (485–488). Further, antitumor vaccines have been developed using plasmid DNA and recombinant viruses that contain antigens fused to phosphatidylserine-binding domains of milk fat globule epidermal growth factor-factor VIII proteins (MFGE8, also known as lactadherin) (489, 490). This protein facilitates the binding of fusion proteins to EVs, making it a potential antitumor vaccine. An ongoing phase I clinical trial tests whether plant exosomes can deliver curcumin to colon tumors (491).

## 7 Nanoparticle-mediated ablation therapies

Challenges still exist in designing and assessing nanoformulation-mediated therapies that focus on CSCs. A prospect to address the constraint in CSC eradication is ablation therapy by means of heat or freezing kills the cancer cells, which causes necrosis and targets CSCs to undergo a cell death pathway (492–494), but was found to be limited due to their possible mutation to non-tumor tissues (495). Li et al. attempted NMATs to deliver uniform heat/freezing (concentrated to projected lesions) exposure to the solid tumor, protecting surrounding healthy tissues (496). An advanced NMAT cancer treatment, for instance, photothermal therapy, has been established and has become advanced against CSCs. PTT involves the killing of CSCs using high temperatures through NIR (493). NMATs are capable of penetrating deeper into regional tumor tissues to destroy CSCs (497). Nanocarriers like gold nanocarriers, carbon nanocarriers, MXenes, and iron oxide magnetic nanocarriers can produce high temperatures to convert absorbed energy into localized heat in tumors (439; 498–502).

### 7.1 Gold nanocarrier coupled with PTT

**Figure 7:**
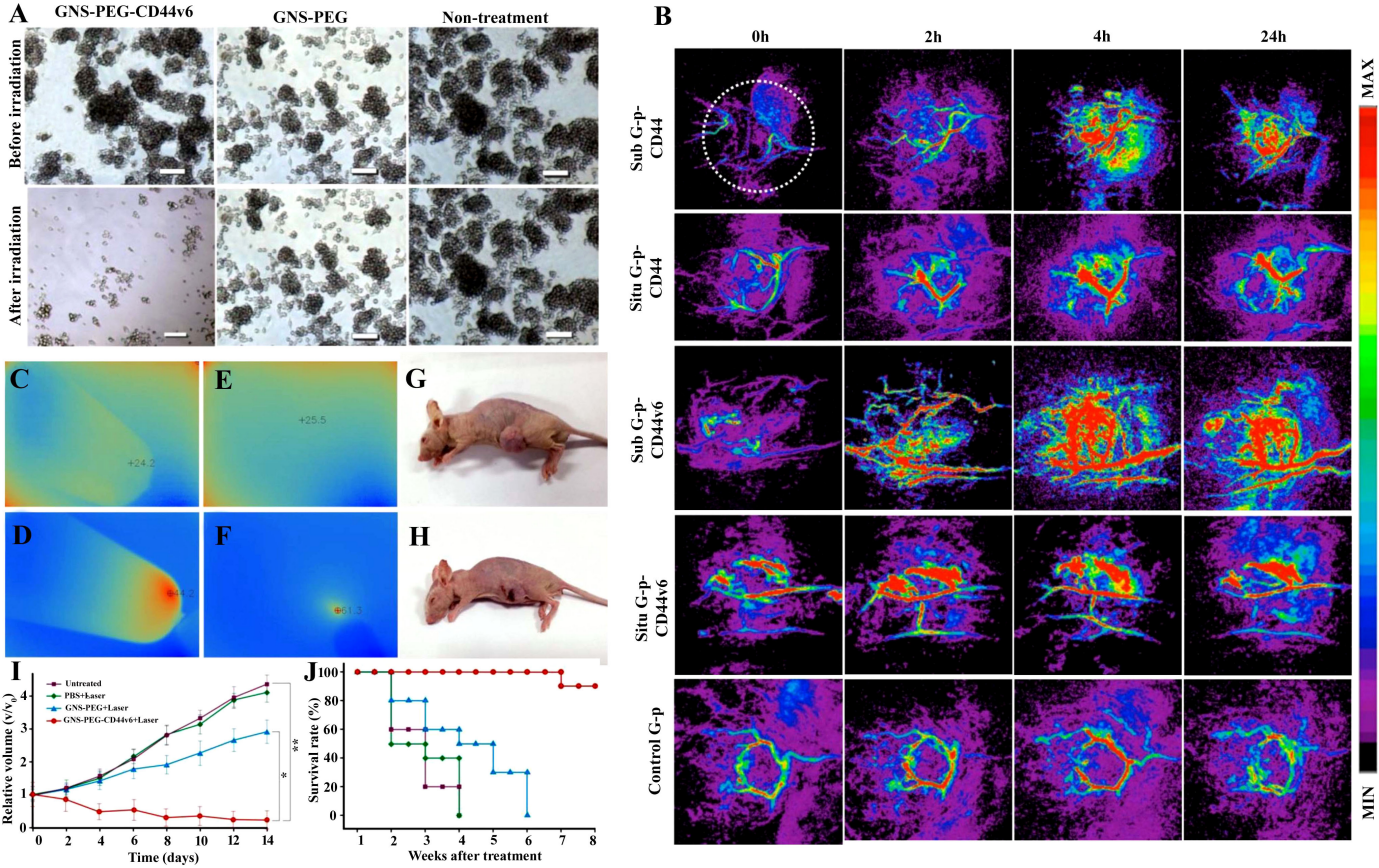
**(A)** A comparison of microscopy descriptions of gastric cancer stem cell (GCSC) spheroid colonies treated with GNS-PEG-CD44v6 and GNS-PEG irradiated for 24 h with near-infrared (NIR) laser (790 nm, 1.5 W/cm^2^, 5 min) showing damaged spheroid colonies in NIR irradiated GNS-PEG-CD44v6. **(B)** Photoacoustic (PA) images attained before and after injection (0, 2, 4, and 24 h) with GNS-PEG-CD44, GNS-PEG-CD44v6 (the first and third row depict a subtumor, while the second and fourth display orthotopic tumor taken as test group), and GNS-PEG (the fifth row denotes a subtumor taken as control). Observation of **(C)** deionized water and **(D)** GNS in a tube exposed to NIR radiation (790 nm, 0.3 W/cm^2^, 3 min) using infrared microscopic imaging. On NIR laser irradiation, subcutaneous GC tumors are shown **(E)** without and **(F)** with injections of GNS-PEG-CD44v6. An injection of GNS-PEG-CD44v6 was administered to a nude mouse of GC subcutaneous xenograft **(G)** with and **(H)** without laser exposure (790 nm, 0.3 W/cm^2^, 3 min). **(I)** Growth curves of GC tumors exposed to NIR laser treatment (790 nm, 0.8 W/cm^2^, 5 min), which were additionally treated with GNS-PEG-CD44v6, GNS-PEG, and PBS, along with the untreated control group. **(J)** Treatment-induced survivability rate (%) was assessed for GC tumor-bearing mice after 8 weeks in comparison to controls. Reused for illustrative purposes with permission from Liang et al. (499) (Copyright 2015, Ivyspring International Publisher, source: https://www.ncbi.nlm.nih.gov/pmc/articles/PMC4493535/).

PTT has been demonstrated using nanospheres, nanocages, nanoshells, nanorods, and nanostars that exhibit surface plasmon resonance (SPR) in the NIR region, hence producing heat (503, 504). The findings of Atkinson et al. (505) on local hyperthermia delivered by Au nanoshells eliminated radio-resistant breast cancer stem cells, resulting in a reduction of tumor size and preventing the increased percentage of ALDH^+^ (505). Rastinehad et al. (506) tested gold nanoshells with PTT on prostate cancer and observed tumor reduction in 94% of patients without side effects (506). Tian et al. (507) fabricated the hollow gold nanospheres with CD271 monoclonal antibody to target osteosarcoma CSCs through PTT, causing cytotoxicity of osteosarcoma CSCs, resulting in apoptosis and DNA double-strand breaks (507). An investigation found that the synergistic combination of PTT and gold nanocages through the recognition of the sigma-2 ligand SV119 has the ability to eradicate breast CSCs (498). Using gold nanostars loaded with retinoic acid (RA) and dendritic polyglycerol (GNS-dPG) with multiple attachment sites of HA is effective in targeting CSCs (508). Liang and colleagues demonstrated that CSCs could be eradicated by means of a gold nanostar-based approach coupled with PTT and when modified with CD44v6 monoclonal antibodies are effective against gastric CSCs (499). The gold nanostar (GNS)-based PEGylated along with CD44v6 monoclonal antibody-conjugated nanoprobes (GNS-PEG-CD44v6, a test group) showed tremendous stability and biocompatibility (499). The investigators tested the synthesized GNS-PEG-CD44v6 (taken as the test group) to selectively eliminate gastric cancer stem cells (GCSCs), for which the CD44^+^-expressing spheroid colonies were incubated with the test group and GNS-PEG (taken as the control group), along with untreated GCSCs for comparison. Laser irradiation (1.5 W/cm^2^) was then applied to all groups for 5 min. The test group showed deteriorated colonies, in contrast to the control and untreated groups, under laser irradiation as represented in **Figure 7A** (499). *In vivo* photoacoustic (PA) imaging of a gastric tumor was carried out using a NIR laser (720 nm) with moderate energy to identify neovascularization and have a high PA contrast effect on the tumor. GNS-PEG-CD44v6 was tested and found to induce a steady upsurge in signal within 4 h. There was a strong signal fortification close to the stomach in subcutaneous tumors, indicating a gradual accumulation of GNS-PEG-CD44v6 and identifying the vascular system. PA images attained before and after injection (0, 2, 4, and 24 h) with GNS-PEG-CD44, GNS-PEG-CD44v6 (the first and third rows depict a subtumor, while the second and fourth display orthotopic tumor), and GNS-PEG (the fifth row denotes a subtumor) are presented in **Figure 7B**. The GNS enhanced the vessel signals and made them accumulate in the perivascular spaces and diffuse into the adjacent tumor tissues after which the signal was found to reduce nearly at 24 h. Correspondingly, GNS-PEG-CD44 doses resulted in parallel agglomeration, but the effects were less pronounced than GNS-PEG-CD44v6 (**Figure 7B**). Control PA signals did not show any robust enhancement, and in intravascular signals, only a slight increment was observed in 2–4 h (**Figure 7B**). Furthermore, GNS-PEG-CD44v6 was tested for its ability to selectively target GCSCs expressing CD44, with high efficiency of photothermal conversion and photothermal ablation (499). GNSs provided an attractive candidate for photothermal agents due to their significant heating capabilities (509, 510), thus overriding CSCs’ resistance to photodynamic therapy and general photothermal treatment. The investigators tested the potential of using GNS-PEG-CD44v6 as a smart imaging probe to detect GCSCs in gastric cancer (GC) using infrared microscopic imaging 499). After GNS-PEG-CD44v6 exposure, subcutaneous tumors from GC xenograft mice were irradiated with NIR lasers. **Figures 7C–F** show that the temperature of the treated tumor site significantly increased within 3 min after laser irradiation, and the color changed from blue to red as demonstrated by infrared imaging. Despite the absence of GNS-PEG-CD44v6 injection or laser irradiation, the infrared imaging signal did not change color, showing no apparent temperature variation when the temperature was increased (**Figure 7E**). In the xenograft mice, the tumor volume was unchanged (**Figure 7G**), while necrotic areas were observed in treated tumor tissues (**Figure 7H**), which may perhaps be due to the sharp structural features of nanostars, making it a more efficient photothermal transducer (511). An analysis of tumor growth curves from four groups after treatment with GNS-PEG-CD44, GNS-PEG, and PBS, respectively, as well as control groups without treatment. As a result of the GNS-PEG-CD44v6 treatment, the tumor volumes of the treated group showed a significant statistical difference and reduced after two weeks of therapy (**Figure 7I**). While GNS-PEG, based on passive targeting therapy, had negligible effect on tumor growth, the untreated groups and PBS groups plus NIR laser did not exhibit any significant therapeutic effect. **Figure 6H** illustrates the survival time of mice treated with GNS-PEG-CD44v6 was significantly longer than in control mice treated with GNS-PEG, PBS, or untreated (**Figure 7J**). This results from the fact that nanoprobes targeting GCSCs can extend tumor-bearing mice's survival time. A new study used aptamers conjugated with gold nanorods to specifically target prostate cancer stem cells in combination with NIR (512). Peng and Wang tailored gold nanorods with anti-CD133 monoclonal antibodies to selectively target and destroy CD133^+^ cells in glioblastoma cell lines in response to the laser beam (513).

### 7.2 Carbon-based NCs with PTT

**Figure 8:**
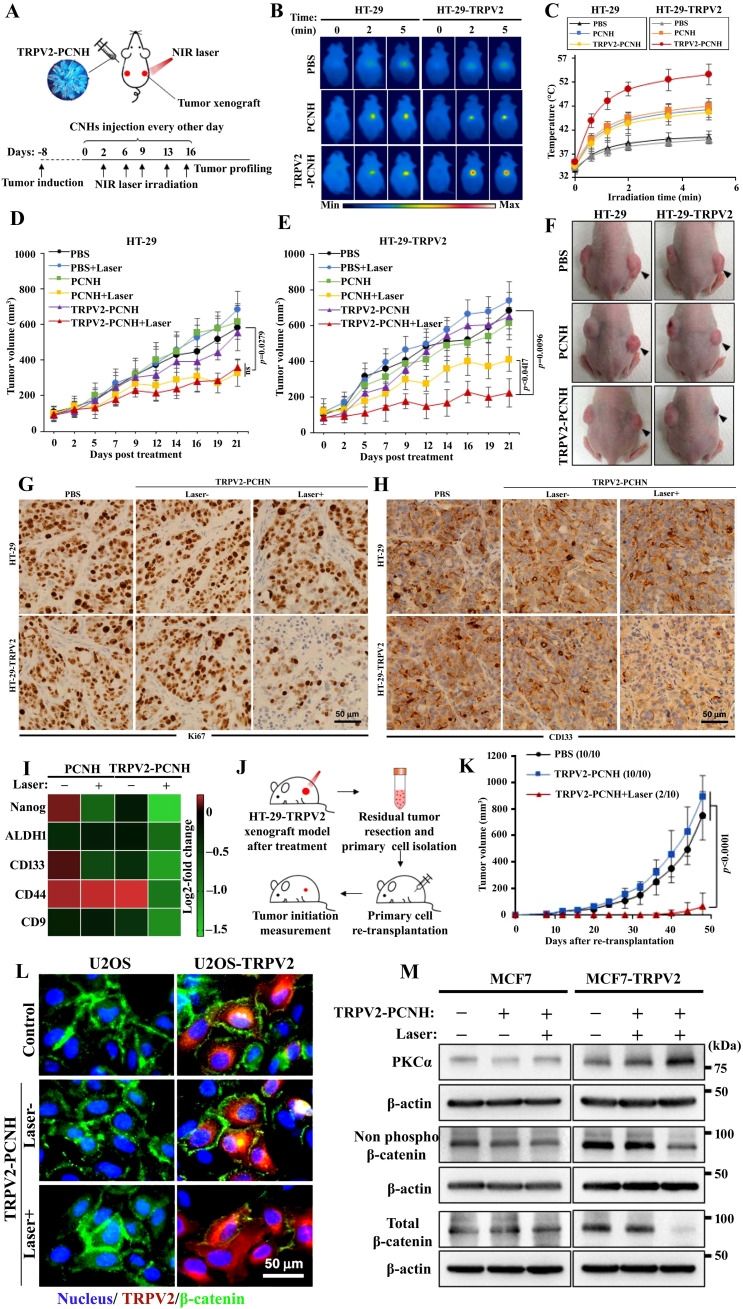
**(A)** A tumor xenograft model was established in mice on day 8 with the inoculation of HT-29 control cells and transfected TRPV2. The treatments PBS, PCHN, and TRPV2–PCNH were administered intraperitoneally (i.p.) on day 0 and administered every other day until day 16. Near-infrared (NIR) laser of 1,064 nm (1 W to 50 mW mm^−2^) was applied for 5 min to the right side of tumor on days 2, 6, 9, 13, and 16. **(B)** A thermographic infrared camera was used to monitor the surface temperature of the body during laser irradiation. **(C)** A laser-induced increase in temperature was observed in mice with HT-29 or HT-29–TRPV2 tumors after nanocomplex injection and measured at 24 **(h)** Tumor volume measured in **(D)** HT-29 and **(E)** HT-29-TRPV2 in different treatment groups with/without NIR laser exposure. A significant reduction in tumor volumes was observed in nude mice bearing HT-29–TRPV2 following TRPV2–PCNH+laser treatment. **(F)** Tumor-bearing nude mice with HT-29 and HT-29–TRPV2 photographed on day 16 (black arrows indicating irradiated tumors). Xenograft models overexpressing TRPV2 and TRPV2–PCNH+laser inhibit tumor reinitiation. Immunohistochemical analysis showed that expressions of **(G)** Ki-67 and **(H)** CD133 expressions were documented to reduce with TRPV2–PCNH+laser in primary tumor sections of xenografts overexpressing TRPV2. **(I)** The RT-qPCR analysis of tumors with TRPV2 overexpression from mice treated with TRPV2–PCNH and laser irradiation exhibited declines in mRNA of stemness-associated markers (Nanog, ALDH1, CD133, CD44, and CD9). **(J)** Methodology followed that of Yu et al. (517) for tumorigenesis experiments involving resection, isolation, re-implantation, and tumor initiation investigation. **(K)** The cells isolated from xenograft tumors showed reduction in tumor proliferation following TRPV2–PCNH+laser treatment in comparison with PBS and TRPV2–PCNH tested groups. **(L)** TRPV2–PCNH+laser irradiation in osteosarcoma cell line (U2OS) demonstrated reduced β-catenin expression as demonstrated by immunostaining images (Hoechst specifies nuclei, mCherry specifies TRPV2, and Alexa488 specifies β-catenin). **(M)** Western blotting illustrated downregulated non-phosphorylated and total β-catenin expression and upregulated PKCα expression in MCF7–TRPV2 cells with TRPV2–PCNH+laser treatment. β-Actin was used as a control for normalization. Reused for illustrative purposes with permission from Yu et al. (517) (Copyright 2020, *Nature Communications*, source: Photothermogenetic inhibition of cancer stemness by near-infrared-light-activatable nanocomplexes - PubMed (nih.gov)).

Carbon-based NCs with robust NIR absorption, thermal conductivity, ease of fabrication, and superior biocompatibility are a great choice for CSC-targeted therapy (514). Wang and colleagues found that carbon nanotubes (CNTs) coupled with CD133 monoclonal antibodies after NIR light exposure potentially reduced self-renewal and tumorigenesis of cancer stem cells in glioblastoma (515). The combination of organoselenium-modified CNTs with PTT successfully destroyed CSCs producing reactive oxygen species, resulting in apoptosis (516). A study reported that PTT with Multiwalled Carbon Nanotube (MWCNTs) eliminated both differentiated cells of tumor and tumor regression of breast cancer stem cells *in vivo* by necrotizing and destroying the CSCs (439). The transient receptor potential vanilloid family type 2 (TRPV2)-PEGylated carbon nanohorn (PCNH) was found to reduce cancer stemness in the presence of NIR irradiation (517) by activating Ca^2+^ influx, hence altering intracellular Ca^2+^ overload, which has been shown to cause apoptosis with TRPV2 overexpression. The human colorectal (HT-29) tumor growth reduction with the laser-driven TRPV2–PCNH was also experimented on nude mice (517), and the results are shown in **Figures 8A–M**. TRPV2–PCNH suppresses cancer stemness when exposed to NIR irradiation, contributing to intracellular Ca^2+^ overload that induces apoptosis (517). As shown in **Figure 5**, the researchers subcutaneously administered tumor xenograft mice with HT-29 colorectal cells or their TRPV2-overexpressing derivatives into their flanks in order to evaluate the effects of anticancer phototherapy. The xenograft mice were divided into groups: PBS as blank control, PBS+laser as laser control, PCNH as non-targeted nanoparticle control, PCNH+laser as non-targeted phototherapy control, TRPV2–PCNH as targeted nanoparticle control, and TRPV2–PCNH+laser as targeted phototherapy groups. Mice of both cell lines (HT-29 or transfected TRPV2 HT-29 cells) were intraperitoneally injected with 5 mg/kg doses of nanocomplexes every other day. Following 24 h of treatment, the mice were subjected to 5-min laser exposure (1 W, ~50 mW mm^−2^) to the right side of the tumor on days 2, 6, 9, 13, and 16 (**Figure 8A**). Thermographic infrared imaging of body surface temperatures was performed during laser irradiation (**Figure 8B**). HT-29–TRPV2 tumors from the group treated with TRPV2–PCNH+laser were the only ones to attain temperature levels above 52°C (activation threshold for TRPV2) (**Figure 8C**). Laser-irradiated PCNH or TRPV2–PCNH NPs reduced the rate of HT-29 (**Figure 8D**) and HT-29-TRPV2 (**Figure 8E**) tumor growth in mice compared with those receiving PBS. TRRV2–PCNH+laser suppressed HT-29–TRPV2 tumors more than any other treatment group, indicating that TRRV2–PCNH targets TRPV2-overexpressing cells selectively. A similar effect was observed in the TRRV2–PCNH+laser group in comparison with non-targeted phototherapy and blank control. HT-29–TRPV2 tumors exposed to laser irradiation were significantly smaller than tumors on the opposite flanks of the same mice without laser irradiation, whereas no effects of laser irradiation on HT-29 xenografts were observed (**Figure 8F**). The ability of laser-driven TRPV2–PCNH nanoparticles to regulate cancer stemness was evaluated via immunohistochemistry staining of Ki-67 and CD133, which are proliferation and stem cell markers, respectively. Laser irradiation resulted in significantly lower expression of both Ki-67 (**Figure 8G**) and CD133 (**Figure 8H**) markers in HT-29-TRPV2 tumor tissues. RT-qPCR investigation showed a decrease in mRNA levels of stemness-associated markers as a result of the laser irradiation effect on mouse tumor tissues tested with PCNH or TRPV2–PCNH NPs (**Figure 8I**). The resection of HT-29–TRPV2 tumors after treatment, following digestion and transplantation into nude mice (**Figure 8J**), resulted in aggressive tumor formation (100%) in non-irradiated tumors in contrast to less tumor growth (20%) in irradiated tumors (**Figure 8K**). The phototherapeutic efficacy of TRPV2–PCNH may improve drug resistance and inhibit cancer stemness. The U2OS osteosarcoma cells overexpressing TRPV2 when receiving TRPV2–PCNH+laser resulted in downregulated β-catenin (associated with carcinogenesis) expression in contrast to the non-laser-treated TRPV2–PCNH and control group (**Figure 8L**). To describe the mechanism, the authors examined laser-induced effects on protein kinase (PKCα), which in combination with Ca^2+^ phosphorylate β-catenin ultimately led to its reduced expression (518). The expression of protein kinase (PKCα) was upregulated in TRPV2-transfected MCF7 (breast cancer) cells following phototherapy revealed through Western blotting expression (**Figure 8M**). The non-phosphorylated and total β-catenin expressions in TRPV2-transfected MCF7 cells were reduced, whereas both protein expression levels were unchanged in MCF7 control (without TRPV2 transfection) (**Figure 8M**). Yu et al. (517) validated that Ca^2+^ influx induced by TRPV2–PCNH+laser stimulates PKCα, leading to downregulated Wnt/β-catenin signaling and related genes.

### 7.3 MXene with PTT

**Figure 9:**
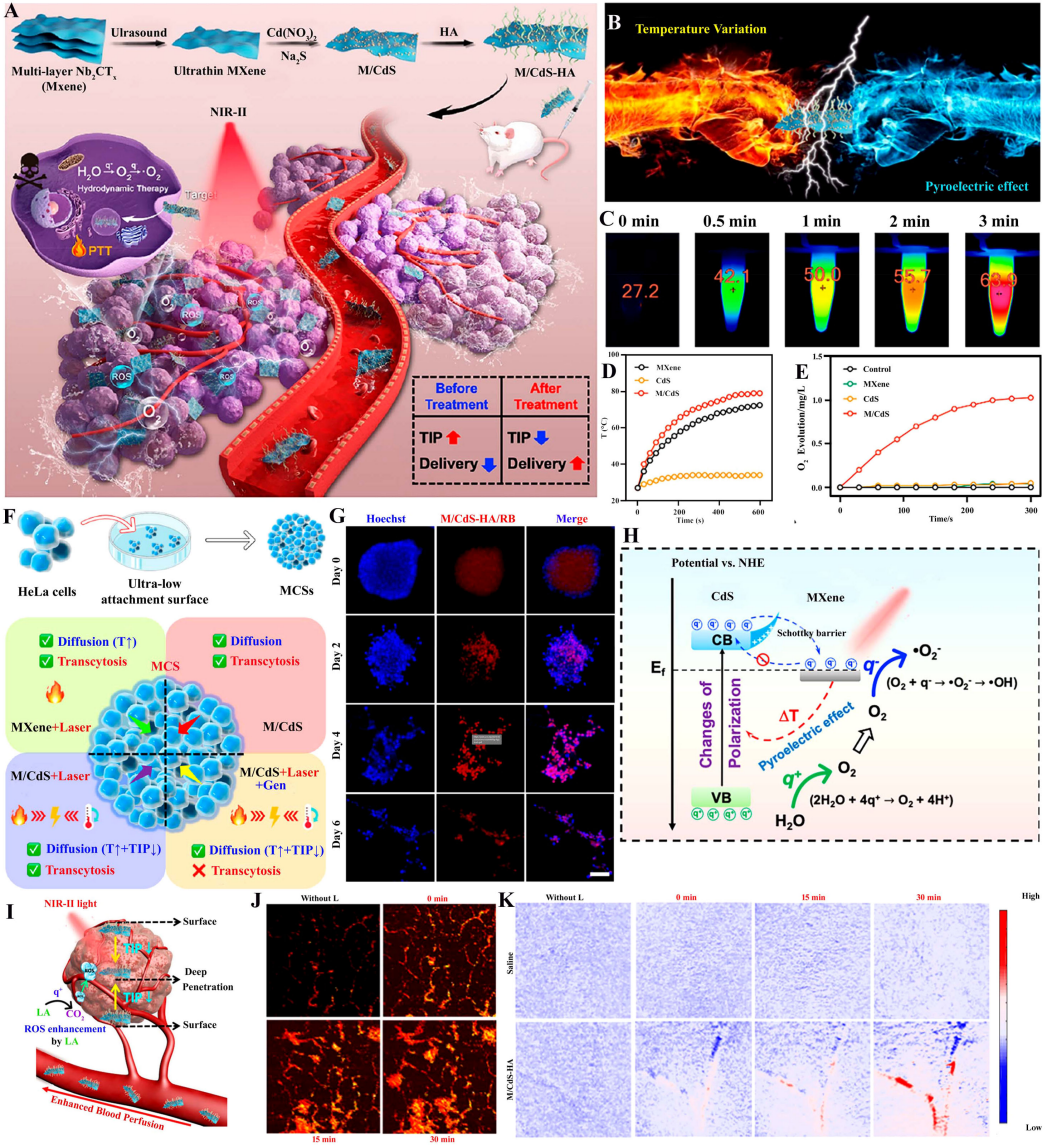
MXene-mediated photothermal ablation of cancer stem cells (CSCs). **(A)** An overview of the synthesis and use of “nano-lymphatic” (M/CdS/HA) for increased tumor penetration, photothermal (NIR-II), and hydrodynamic therapies with the decrease in tumor interstitial pressure (TIP) via pyroelectric catalysis. **(B)** Pyroelectric effect with temperature variation depicted schematically. **(C)** The infrared thermal image (ITI) demonstrated the excellent photothermal effect of M/CdS under laser irradiation at 3 min and could confirm further usage for both PTT and pyroelectric catalysis (here, 0 min was considered as control). **(D)** Photothermal consequence of CdS, MXene, and MXene/CdS. **(E)** The curves of O_2_ generation due to CdS, MXene, and M/CdS under laser irradiation (1,064 nm, 1.0 W/cm^2^) in comparison to control. **(F)** The illustration of multicellular spheroid (MCS) formation and the penetration mechanism showing increased diffusion with rise in temperature (T↑), reduction in TIP, and transcytosis in MXene+laser, MXene/CdS, MXene/CdS+laser, and MXene/CdS+laser+genistein (Gen) treatments. **(G)** Confocal fluorescence imaging of MCSs on exposure with MXene/CdS-HA/RB with NIR-II laser irradiation (1,064 nm) from day 0 to 6 (scale bar = 100 μm). **(H)** Representation of pyroelectric catalysis for water splitting and induced reactive oxygen species (ROS) production in case of treatment. **(I)** Enhanced tumor penetration with improved blood perfusion due to TIP reduction and induced ROS generation due to lactic acid (LA) in the tumor. **(J)** MXene/CdS-HA treatment of tumor blood vessels with/without 1,064-nm NIR-II laser exposure at various time intervals (0, 15, and 30 min) was visualized using photoacoustic illustration. **(K)** Concentration of oxygen in tumor blood vessels treated with saline as a control and MXene/CdS-HA (with/without 2 min of 1,064-nm irradiation exposure) at various time intervals of 0, 15, and 30 min. Reused for illustrative purposes with permission from He et al. (502) (Copyright 2021, American Chemical Society, source: Pyroelectric Catalysis-Based “Nano-Lymphatic” Reduces Tumor Interstitial Pressure for Enhanced Penetration and Hydrodynamic TherapyACS Nano).

MXenes are 2D layered transition metal carbides with nitrides or carbonitrides and display robust absorption of NIR beam, causing hyperpyrexia in order to ablate tumors efficiently (519, 520). *In situ* growth of CdS on an ultrathin Nb2C nanosheet (MXene) produces M/CdS, which is then modified with CSCs targeting HA to form the nano-lymphatic (M/CdS-HA) material (502). HA-mediated tumor targeting and NIR-II (1,064 nm) laser irradiation targets the “nano-lymphatic” toward the tumor region, which reduces the tumor interstitial pressure (TISP) via PTT (**Figure 9A**). The tumor interstitial fluid pressure (TIFP) is decreased as a result of the temperature variation, prompting CdS to decompose the tumor interstitial fluid through pyroelectric catalysis. As a result of the reduction in TISP and TIFP, the “nano-lymphatic” penetrates deeper into tumors; at the same time, pyroelectric catalysis generates ROS in deep tumor stem cells, leading to apoptosis and necrosis due to oxidative damage. **Figure 9B** illustrates the pyroelectric effect and the relationship between temperature variation and pyroelectric current, whereby the motion of atoms in the pyroelectric material is influenced by temperature variation, leading to the change in polarization for a pyroelectric field integrated into it. This PTT and pyroelectric catalysis of M/CdS was visually illustrated via infrared thermal imaging (ITI), which demonstrated the exceptional result under laser irradiation at 3 min (**Figure 9C**) (502). The PTT effect, pyroelectric current, and potential response under 1,064-nm NIR-II irradiation of CdS, MXene, and MXene/CdS were evaluated and compared (**Figure 9D**). MXene and MXene/CdS displayed a substantial increase in temperature (ΔT = 45°C and 52°C) in 10 min compared to CdS (**Figure 9D**). Unlike MXene and CdS, M/CdS could produce significant amounts of O_2_ with 5 min of 1,064-nm laser irradiation, which showed water splitting by means of pyroelectric catalysis (**Figure 9E**) (502). Multicellular spheroids (MCSs) were formed with HeLa cells grown into a culture dish with an ultralow attachment surface. MCSs were previously treated with genistein (Gen), an endocytosis inhibitor, to interrupt the passage of M/CdS across the cell monolayer to study the relationship between diffusion and transcytosis (**Figure 9F**). The structural changes of MCSs with laser irradiation from day 0 to day 6 of MXene/CdS-HA/RB were first demonstrated by fluorescent imaging, as shown in **Figure 9G**. Blue fluorescence indicates the nucleus, while red fluorescence indicates the center of the MCSs, suggesting that MXene/CdS-HA was incorporated into MCSs on day 0 after laser irradiation. Upon laser irradiation, MCSs collapsed progressively on days 2, 4, and 6, indicating deep damage. The pyroelectric catalysis mechanism is presented in **Figure 9H**. MXene plasmon resonance and excitation of CdS were introduced in response to the NIR-II laser due to its large energy gap. Pyroelectric catalysis-based water splitting produced reactive oxygen species from the developed negative and positive oxygen species (**Figure 9H**). Since CdS and MXene have different Fermi levels (Ef) and work functions, the negative charges of CdS are transferred to MXene to equilibrate their Ef. Schottky barriers are formed when the energy band of CdS (n-type semiconductor) is bent upward during equilibrium (521). The light was converted into hyperpyrexia by MXene in combination with laser irradiation. The temperature variation due to the pyroelectric effect directed the negative charge of CdS to move along the Schottky junction toward MXene, prohibiting the backflow of negative charges (496). MXene could also be used as a cocatalyst to improve the catalytic efficiency of CdS. MXene could be used as a cocatalyst to enhance the catalytic efficiency of CdS. The excited negative charge was found to react with O_2_ and generated superoxide (^•^O^2−^) and hydroxyl (^•^OH) radicals, and the positive charge reacted with H_2_O to produce O_2_ and H^+^ as shown in **Figure 9H** (502). Lactic acid (LA) presented enhanced catalytic activity of laser-induced M/CdS-HA and antitumor efficacy (**Figure 9I**). As a result of LA overexpression in the tumor microenvironment, pyroelectric catalysis is prevented from interacting with positive charges, thus increasing ROS production. MXene/CdS-HA treatment of tumor blood vessels with/without 1,064-nm NIR-II laser exposure at various time intervals (0, 15, and 30 min) was visualized using photoacoustic illustration in **Figure 9J**. After 24 injections of M/CdS-HA, the blood perfusion remained very low without laser irradiation. The blood perfusion was enhanced with increasing time of laser exposure as a result of a decrease in TIP (502). Enhanced drug delivery from the blood to the tumor could be achieved by reducing the pressure difference between blood and tumor interstitial fluid, providing an effective force for the delivery of drugs from blood into bulk tumors. A white arrow indicates bleeding spots, which resulted from enhanced blood flow and hyperpyrexia-induced damage; at the same time, the enhanced blood circulation mediated by M/CdS-HA+L could increase the intratumoral oxygen (O_2_) content, improving hydrodynamic therapy (**Figure 9K**). The saline group was shown to exhibit hypoxia on irradiation with no significant change. M/CdS-HA+L exhibited an increment in O_2_ content and contributed to the increased blood perfusion (**Figure 9K**), which ultimately carried the nanomedicine to the tumor site. By regulating TIP, nanomedicine could effectively penetrate deeper into tumors, and the ROS resulting from the pyroelectric catalysis could further damage deep tumor stem cells (517).

## 8 Conclusion

Current cancer treatment failures are thought to be rooted in CSCs, which are vastly resistant to conventional therapies, leading to recurrence and metastasis. A significant amount of investigation has promoted the practice of NCs for cancer therapy without targeting CSCs. Our review describes various functionalized NCs, EVs, and PTT mediated for improving CSC ablation. A major challenge in clinical translation research for specifically targeting CSCs with modified NCs and its outcome depends on factors involving specificity and protection. Developing clinical applications of modified NCs against CSCs requires multidisciplinary collaboration, as well as continuous basic and applied research aimed at understanding their properties. A state-of-the-art nanotechnology approach will also be required to develop more effective strategies for eradicating CSCs. It is very likely that the NCs targeting CSCs will attain efficacious clinical translation in the upcoming days, allowing patients to benefit from unique treatments.

## Glossary

ABC transporters: ATP binding cassette transporters
ABCG2: ATP-binding cassette super-family G member 2
ALA: 5-aminolevulinic acid
ALDH: aldehyde dehydrogenase
ALDH1A1: aldehyde dehydrogenase 1 family, member A1
ALL: lymphoblastic leukemia
AML: acute myeloid leukemia
ANA: antinuclear antibodies
Antho-NPs: anthothecol-encapsulated PLGA nanoparticles
ATRA: all-*trans*-retinoic acid
AuNPs: gold nanoparticles
BTZ: bortezomib
CAP: cold atmospheric plasma
CAR T cells: chimeric antigen receptor T cells
Cas9: CRISPR-associated protein 9
CD: cluster of differentiation
CDDP: cisplatin (*cis*-diamminedichloroplatinum)
CDF: 3,4-difluorobenzylidene curcumin
CdTe-QDs: cadmium telluride quantum dots
CHO/CHOL: cholesterol
CNTs: carbon nanotubes
COPA: PLA-chitosan-PEG-folic acid
COPAB: COPA and COPB
COPB: PLA-chitosan-PEG-glucose
CPT: camptothecin
CRISPR: Clustered Regularly Interspaced Short Palindromic Repeats
CSCs: cancer stem cells
CTAB: cetyltrimethylammonium bromide
CXCR4: C-X-C chemokine receptor type 4
DAPT: *N*-[*N*-(3,5-difluorophenacetyl)-{{sc}}l{{/sc}}-alanyl]-*S*-phenylglycine *t*-butyl ester
Dex: dexamethasone
DLin-MC3-DMA: (6*Z*,9*Z*,28*Z*,31*Z*)-heptatriaconta-6,9,28,31-tetraen-19-yl-4-(dimethylamino)butanoate
DMPC: dimyristoyl phosphatidylcholine
DNA: deoxyribonucleic acid
DOPC: dioleoyl phosphatidylcholine
DOPE: dioleoyl phosphatidylethanolamine
DOTAP: dioleoyl trimethylammonium-propane
DOX: doxorubicin
DP-CLPs: dual-modified cationic liposomes
DPPC: dipalmitoyl phosphatidylcholine
DPPG: dipalmitoyl phosphatidylglycerol
DSPC: distearoyl phosphatidylcholine
DSPG: distearoyl phosphatidylglycerol
DTX: docetaxel
DX: dexamethasone (Dex)-associated liposomes
EAC: Ehrlich ascites carcinoma
ECM: extracellular matrix
EGFR: epidermal growth factor receptor
EMA: European Medicines Agency
EMT: epithelial–mesenchymal transition
EPC: ethyl phosphatidylcholine
EpCAM: epithelial cellular adhesion molecule
EPG: ethyl phosphatidylglycerol
EPR: enhanced permeability and retention
ESA: epithelial‐specific antigen
ESCC: esophageal squamous cell carcinoma
EVs: extracellular vesicles
FA: folic acid
FDA: Food and Drug Administration
FIH: first in human
GBM: glioblastoma multiforme
Gem: gemcitabine
Gen: genistein
GQDs: graphene quantum dots
HA: hyaluronic acid
HCC: hepatocellular carcinoma
HER2: human epidermal growth factor receptor 2
Hh: Hedgehog
HIV: human immunodeficiency virus
HNSCC: head and neck squamous cell carcinoma
HPV: human papillomavirus
HSP: heat shock proteins
HSPC: fully hydrogenated phosphatidylcholine
HT: human colorectal
IHC: immunohistochemistry
IL-2: interleukin-2
ILVs: intraluminal vesicles
IU: investigational use
KLF4: Krüppel-like factor 4
LA: lactic acid
LF: liposomal formulation
LGR5: leucine-rich repeat-containing G protein-coupled receptor 5
LiCl: lithium chloride
M/CdS: MXene-cadmium sulfide
mAbs: monoclonal antibodies
MAGE A3: melanoma antigen family A3
MCS: multicellular spheroid
MDR: multidrug resistance
MEK: mitogen-activated extracellular signal-regulated kinase
MH: 6-mercapto-1-hexanol
MHCC97H: metastatic hepatocellular carcinoma
miRNA-34: microRNA-34
MM: multiple melanoma
MMPs: matrix metalloproteinases
mPEG-*b*-PLA: methoxy-poly(ethylene glycol)-block-polylactic acid
MPEG-DSPE: *N*-(carbonyl-methoxy(polyethylene glycol)-2000)-1,2-distearoyl-*sn*-glycero-3-phosphoethanolamine sodium salt
MSPC: monostearoyl phosphatidylcholine
MTD: maximum tolerable dose
MTX: methotrexate
MUC1: mucin 1
MVBs: multivesicular bodies
MWCNTs: Multiwalled carbon nanotubes
NAP: naproxen
NDD: nanomaterial-loaded drug delivery
NDs: nanodiamonds
NIR: near-infrared
NK cells: natural killer cells
NMATs: nanoparticle-mediated ablation therapies
NPC: nasopharyngeal
NPs: nanoparticles
NSCLC: non-small-cell lung cancer
OCT4: octamer-binding transcription factor 4
PAMAM: polyamidoamine
PARP: poly ADP-ribose polymerase
PBS: phosphate-buffered saline
PC: phosphatidylcholine
PCNH: PEGylated carbon nanohorn
PD-1: programmed cell death protein-1
PDT: photodynamic therapy
PEA: poly(ester amine)
PEG: poly(ethylene glycol)
PEG2000-C-DMG: α-(30-{[1,2-di(myristyloxy)propanoxy]carbonylamino}propyl)-ω-methoxy polyoxyethylene
PEG2000-DSPE: polyethylene glycol 2000-distearoyl phosphatidylethanolamine
PEHAM: polyglycerol, poly(etherhydroxylamine)
Phen: phenformin
PLA: polylactic acid
PLGA: poly(lactic-co-glycolic acid)
PNPs: polymeric nanoparticles
PEG: poly(ethylene glycol)-*b*-poly({{sc}}d{{/sc}},{{sc}}l{{/sc}}-lactide)
PPI: poly(propylene imine)
PpIX: protoporphyrin IX
PS-*b*-PEO: poly(styrene-*b*-ethylene oxide)
PSiNPs: porous silicon nanoparticles
PSMA: prostate-specific membrane antigen
PTT: photothermal therapy
PTX: paclitaxel
QD: quantum dots
RA: retinoic acid
RBCs: red blood cells
RCC: renal cell carcinoma
RNA: ribonucleic acid
ROS: reactive oxygen species
SA: streptavidin
SAL: salinomycin
SALL4: Sal-like protein 4
SAL-NP: salinomycin-loaded PEGylated poly(lactic-*co*-glycolic acid) nanoparticles
SCLC: small-cell lung cancer
siRNA: small interfering RNA
SMO: smoothened inhibitors
SOX2: (sex-determining region Y)-box 2
SPR: surface plasmon resonance
SWCNT: single-walled carbon nanotube
TCR: T-cell receptor
TGF-β: transforming growth factor beta
TIP: tumor interstitial pressure
TMZ: temozolomide
TNBC: triple-negative breast cancer
TNRPV2: transient receptor potential vanilloid family type 2
